# Sulfur Vacancy and Ti_3_C_2_T*_x_* Cocatalyst Synergistically Boosting Interfacial Charge Transfer in 2D/2D Ti_3_C_2_T*_x_*/ZnIn_2_S_4_ Heterostructure for Enhanced Photocatalytic Hydrogen Evolution

**DOI:** 10.1002/advs.202103715

**Published:** 2021-11-21

**Authors:** Tongming Su, Chengzheng Men, Liuyun Chen, Bingxian Chu, Xuan Luo, Hongbing Ji, Jianhua Chen, Zuzeng Qin

**Affiliations:** ^1^ School of Chemistry and Chemical Engineering Guangxi University Nanning 530004 P. R. China; ^2^ Fine Chemical Industry Research Institute School of Chemistry Sun Yat‐sen University Guangzhou 510275 P. R. China; ^3^ School of Resources Environment, and Materials Guangxi University Nanning 530004 P. R. China

**Keywords:** 2D, MXene, photocatalytic, S vacancy, ZnIn_2_S_4_

## Abstract

Constructing an efficient photoelectron transfer channel to promote the charge carrier separation is a great challenge for enhancing photocatalytic hydrogen evolution from water. In this work, an ultrathin 2D/2D Ti_3_C_2_T*_x_*/ZnIn_2_S_4_ heterostructure is rationally designed by coupling the ultrathin ZnIn_2_S_4_ with few‐layered Ti_3_C_2_T*_x_* via the electrostatic self‐assembly strategy. The 2D/2D Ti_3_C_2_T*_x_*/ZnIn_2_S_4_ heterostructure possesses larger contact area and strong electronic interaction to promote the charge carrier transfer at the interface, and the sulfur vacancy on the ZnIn_2_S_4_ acting as the electron trap further enhances the separation of the photoinduced electrons and holes. As a consequence, the optimal 2D/2D Ti_3_C_2_T*_x_*/ZnIn_2_S_4_ composite exhibits a high photocatalytic hydrogen evolution rate of 148.4 µmol h^−1^, which is 3.6 times and 9.2 times higher than that of ZnIn_2_S_4_ nanosheet and flower‐like ZnIn_2_S_4_, respectively. Moreover, the stability of the ZnIn_2_S_4_ is significantly improved after coupling with the few‐layered Ti_3_C_2_T*_x_*. The characterizations and density functional theory calculation demonstrate that the synergistic effect of the sulfur vacancy and Ti_3_C_2_T*_x_* cocatalyst can greatly promote the electrons transfer from ZnIn_2_S_4_ to Ti_3_C_2_T*_x_* and the separation of photogenerated charge carriers, thus enhancing the photocatalytic hydrogen evolution from water.

## Graphical abstract

2D/2D Ti_3_C_2_T*_x_*/ZnIn_2_S_4_ heterojunction is constructed to achieve high‐efficiency photocatalytic hydrogen evolution under visible light. The synergistic effect of sulfur vacancies and Ti_3_C_2_T*_x_* cocatalyst promotes the efficient transfer of photogenerated electrons at the 2D/2D interface, thus greatly enhances the photocatalytic hydrogen evolution performance of 2D/2D Ti_3_C_2_T*_x_*/ZnIn_2_S_4_ composites.

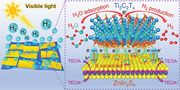

## 1 Introduction

Photocatalytic water splitting, which can directly convert the inexhaustible solar energy into clean and high‐energy‐density hydrogen, is regarded as one of the important ways to solve the global energy shortage and environmental pollution problem.^[^ ^1^ ^]^ Over the past decades, the emergence of novel catalytic materials from particles to nanoscale has promoted the continuous development in photocatalytic water splitting.^[^ ^2^ ^]^ However, due to the limitations of the electronic structure,^[^ ^3^ ^]^ morphology,^[^ ^4^ ^]^ energy band structure,^[^ ^5^ ^]^ and surface chemical state,^[^ ^6^ ^]^ the development of semiconductors with excellent efficiency of photocatalytic water splitting remains a challenge.^[^ ^7^ ^]^ In recent years, ultrathin 2D materials have become promising catalysts for photocatalytic hydrogen reaction due to their advantages of more exposed active sites^[^ ^8^ ^]^ and shorter electron migration distance.^[^ ^9^ ^]^ Among them, nontoxic hexagonal ZnIn_2_S_4_, due to its narrow bandgap and the S–Zn–S–In–S–In–S‐type lamellar stack structure, has attracted great attention in the family of 2D photocatalyst.^[^ ^10^ ^]^ However, most of the reported ZnIn_2_S_4_ was prepared by hydrothermal or solvothermal methods, leading to lamellar cross‐linking and agglomeration, and the active sites cannot fully contact water molecules, such as the classic marigold structure.^[^ ^11^ ^]^ Yang et al. reported that the preparation of highly dispersed ZnIn_2_S_4_ colloidal solution by the refluxing and stripping methods shortened the carrier transport path and effectively inhibited the recombination of electrons and holes.^[^ ^12^ ^]^ The light absorption capacity of 2D ZnIn_2_S_4_, the excitation, migration, and the recombination of photogenerated electron holes in 2D ZnIn_2_S_4_ can be controlled by lattice and outer valence electrons in the nanoscale.^[^ ^13^ ^]^ Therefore, it is beneficial to improve the photocatalytic performance of ZnIn_2_S_4_ by accurately regulating the electronic structure by overcoming the van der Waals force between nanosheets.

Introducing sulfur (S) vacancy into the lattice of ultrathin 2D semiconductors is an effective strategy to tune their electronic structure.^[^ ^14^ ^]^ The vacancy affects the intrinsic bandgap value of semiconductors^[^ ^15^ ^]^ and induces the generation of intermediate energy levels to increase the charge carrier concentration and captures electrons to promote photogenerated carrier separation.^[^ ^16^ ^]^ Recently, Cao et al. reported that the direction of photogenerated electron transfer could be controlled by adjusting the vacancy type of CdS.^[^ ^17^ ^]^ Gao et al. grew S‐vacancy ZnS vertically on the Zn‐In‐LDH surface, which stimulated the synergistic effect of vacancy and 2D interface and improved the performance of photocatalytic hydrogen production.^[^ ^18^ ^]^ However, due to the instability and the limited number of vacancies, 2D semiconductor photocatalysts are still faced with the significant recombination of the photogenerated electrons and holes.^[^ ^19^ ^]^ The introduction of effective cocatalysts to 2D photocatalyst is an effective means to optimize the electron transport path and promote the transfer and separation of photoinduced charge carriers.

Since the first reported MXene in 2011,^[^ ^20^ ^]^ 2D MXenes material has demonstrated excellent performance in the fields of supercapacitor,^[^ ^21^ ^]^ lithium‐ion battery,^[^ ^22^ ^]^ catalysis,^[^ ^23^ ^]^ electromagnetic shielding, and other fields.^[^ ^24^ ^]^ Due to the excellent conductivity,^[^ ^25^ ^]^ hydrophilicity,^[^ ^26^ ^]^ large ultrathin 2D interface,^[^ ^27^ ^]^ a large number of active sites on the surface, and a superior Fermi level position,^[^ ^28^ ^]^ MXenes are widely used as cocatalyst in the field of photocatalytic water splitting.^[^ ^29^ ^]^ For example, the Ti_3_C_2_ MXene was demonstrated as a potential cocatalyst to replace the rare precious metal Pt, the apparent quantum efficiency of Ti_3_C_2_/CdS at 420 nm under visible light reached 40.1% when with Ti_3_C_2_ as the cocatalyst.^[^ ^30^ ^]^ Moreover, Xie et al. found that Ti_3_C_2_T*_x_* wondrously delayed the photocorrosion of sulfide by photogenerated holes through the adsorption of spillage metal ions.^[^ ^31^ ^]^ In recent years, the in situ growth of the photocatalysts on the 2D interface of Ti_3_C_2_ has been widely used for the design of photocatalytic heterosystem. For example, ZnIn_2_S_4_ nanosheets were in situ grown on the surface of Ti_3_C_2_ to improve photocatalytic H_2_ evolution performance.^[^ ^32^ ^]^ However, the analogous vertical contact between the edges of ZnIn_2_S_4_ layer and the Ti_3_C_2_ plane results in a long carrier transfer path, which is not beneficial for the rapid transfer and separation of the electrons and holes, and the S vacancy on the ZnIn_2_S_4_ surface was not taken into account. To further improve the transfer rate of the photoinduced electrons, the rational design of 2D/2D MXene‐based heterostructure with close contact interface and multiactive sites opens up an effective strategy for the construction of efficient photocatalyst for hydrogen production.

Herein, ultrathin 2D ZnIn_2_S_4_ nanosheet containing S vacancy was prepared, and the 2D ZnIn_2_S_4_ was combined with the few‐layered Ti_3_C_2_T*_x_* nanosheet by electrostatic self‐assembly to synthesize the tightly contacted 2D/2D Ti_3_C_2_T*_x_*/ZnIn_2_S_4_ composites, which was used as the efficient photocatalyst for photocatalytic hydrogen evolution. The S vacancy acted as electron traps on the 2D ZnIn_2_S_4_ surface, and the few‐layered Ti_3_C_2_T*_x_* with larger work function can quickly capture the photogenerated electrons on the 2D ZnIn_2_S_4_ nanosheet, which greatly promotes the electron transfer and the separation of photogenerated charge carriers. As a result, the 2D/2D Ti_3_C_2_T*_x_*/ZnIn_2_S_4_ showed a superior photocatalytic hydrogen evolution performance, due to the compact 2D/2D heterointerfaces and the synergistic effect of the S vacancy and the Ti_3_C_2_T*_x_* cocatalyst.

## 2 Results and Discussion

### 2.1 Synthesis, Structure, and Morphology

**Figure 1:**
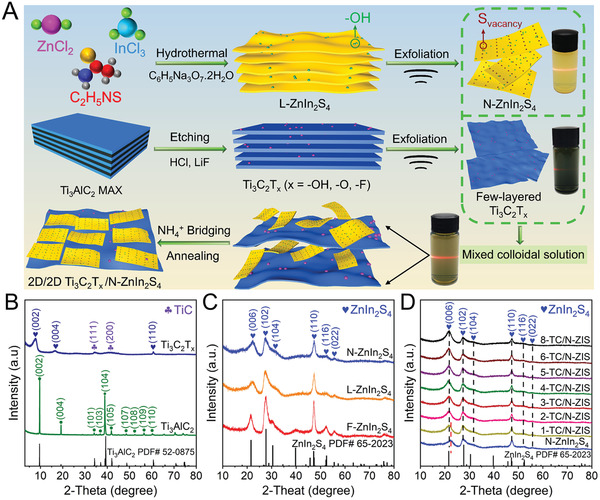
A) Schematic illustration of the synthetic route of L‐ZnIn_2_S_4_, N‐ZnIn_2_S_4_, Ti_3_C_2_T*_x_*, and TC/N‐ZIS composite. XRD patterns of B) Ti_3_AlC_2_, Ti_3_C_2_T*_x_*, C) F‐ZnIn_2_S_4_, L‐ZnIn_2_S_4_, N‐ZnIn_2_S_4_, and D) *x*‐TC/N‐ZIS (*x* = 1, 2, 3,4, 5, 6, and 8).

**Figure** **1**A illustrates the synthetic route of layered ZnIn_2_S_4_ (labeled as L‐ZnIn_2_S_4_), ZnIn_2_S_4_ nanosheet (labeled as N‐ZnIn_2_S_4_), few‐layered Ti_3_C_2_T*_x_*, and Ti_3_C_2_T_x_/N‐ZnIn_2_S_4_ (labeled as TC/N‐ZIS) composites. The TC/N‐ZIS with different amounts (1, 2, 3, 4, 5, 6, and 8 wt%) of Ti_3_C_2_T_x_ was denoted as 1‐TC/N‐ZIS, 2‐TC/N‐ZIS, 3‐TC/N‐ZIS, 4‐TC/N‐ZIS, 5‐TC/N‐ZIS, 6‐TC/N‐ZIS, and 8‐TC/N‐ZIS, respectively. The ZnIn_2_S_4_ nanoflower (labeled as F‐ZnIn_2_S_4_) sample was simply synthesized by a hydrothermal method (Figure S1, Supporting Information). Compared to the synthesis condition of F‐ZnIn_2_S_4_, the L‐ZnIn_2_S_4_ was obtained by adding additional trisodium citrate dihydrate to the reaction solution before hydrothermal reaction. In this way, ZnIn_2_S_4_ was grown in layers along the plane direction. Therefore, N‐ZnIn_2_S_4_ with rich S vacancy can be simply prepared by treating the L‐ZnIn_2_S_4_ with ultrasonication self‐exfoliation due to the weak Van der Waals force between the ZnIn_2_S_4_ layers.^[^ ^19^ ^]^ The multilayer Ti_3_C_2_T*_x_* was prepared by etching the Ti_3_AlC_2_ with HCl/LiF solution as the etchant, and the few‐layered Ti_3_C_2_T*_x_* can be obtained by exfoliating the multilayer Ti_3_C_2_T*_x_* with ultrasonication. Subsequently, the TC/N‐ZIS was obtained by coupling the N‐ZnIn_2_S_4_ with the few‐layered Ti_3_C_2_T*_x_* nanosheet through the electrostatic self‐assembly strategy with the assistance of the NH_4_^+^.

From the SEM images of F‐ZnIn_2_S_4_ sample (Figure S2A,D, Supporting Information), F‐ZnIn_2_S_4_ showed a tightly packed marigold‐like morphology, which in accordance with the reported results.^[^ ^33^ ^]^ However, the L‐ZnIn_2_S_4_ exhibited the lamellar structure (Figure S2B,E, Supporting Information). The Ti_3_AlC_2_ also showed the lamellar structure (Figure S2C, Supporting Information), and the multilayer Ti_3_C_2_T*_x_* obtained by etching the Ti_3_AlC_2_ displayed the accordion‐like morphology (Figure S2F, Supporting Information).

The L‐ZnIn_2_S_4_ can be completely exfoliated into light yellow N‐ZnIn_2_S_4_ colloidal solution by short‐time ultrasonic treatment in the absence of any intercalator or surfactant species (Figure S3, Supporting Information), while the F‐ZnIn_2_S_4_ cannot be completely dispersed in the solution under ultrasonic treatment for 3 h and will precipitate after standing for 1 h (Figure S4, Supporting Information). To figure out the reason why the N‐ZnIn_2_S_4_ can stably exist in the solution, the zeta potential of N‐ZnIn_2_S_4_ colloidal solution was measured (Figure S5, Supporting Information). The high negative charge value of −39.8 mV of the N‐ZnIn_2_S_4_ colloidal solution indicated that there was a strong repulsive force between the N‐ZnIn_2_S_4_ nanosheets,^[^ ^34^ ^]^ which accounts for the stability of the N‐ZnIn_2_S_4_ colloidal solution.

In addition, with the –OH, –O, and –F functional groups on the surface of Ti_3_C_2_T*_x_*, the zeta potential of few‐layered Ti_3_C_2_T*_x_* colloidal solution was measured to be −31.8 mV. Therefore, the Ti_3_C_2_T*_x_* nanosheets were electronegative and exhibited excellent dispersity in water.^[^ ^35^ ^]^ Due to the electronegative surface of the Ti_3_C_2_T*_x_* and the N‐ZnIn_2_S_4_, a green and transparent mixed Ti_3_C_2_T*_x_*/N‐ZnIn_2_S_4_ colloidal solution can be formed when 1.36 mL Ti_3_C_2_T*_x_* colloidal solution (1.5 mg mL^−1^) was added to 50 mL N‐ZnIn_2_S_4_ colloidal solution (2.0 mg mL^−1^), and the zeta potential was changed to −39.4 mV. Moreover, with the increased amount of Ti_3_C_2_T*_x_* colloidal solution from 1.36 to 5.80 mL, the zeta potential of the mixed Ti_3_C_2_T*_x_*/N‐ZnIn_2_S_4_ colloidal solution increased from −39.8 to −36.2 mV. Hence, when the NH_4_^+^ was induced to the mixed Ti_3_C_2_T*_x_*/N‐ZnIn_2_S_4_ colloidal solution by gradually adding NH_4_HCO_3_ solution, the N‐ZnIn_2_S_4_ and Ti_3_C_2_T*_x_* were drawn together and formed the 2D/2D Ti_3_C_2_T*_x_*/N‐ZnIn_2_S_4_ composite (Figure S6, Supporting Information). Notably, the NH_4_^+^ here acted as a binder to attract the N‐ZnIn_2_S_4_ nanosheet and the Ti_3_C_2_T*_x_* to contact with each other and form a compact heterostructure at the Ti_3_C_2_T*_x_*/N‐ZnIn_2_S_4_ interfaces.

**Figure 2:**
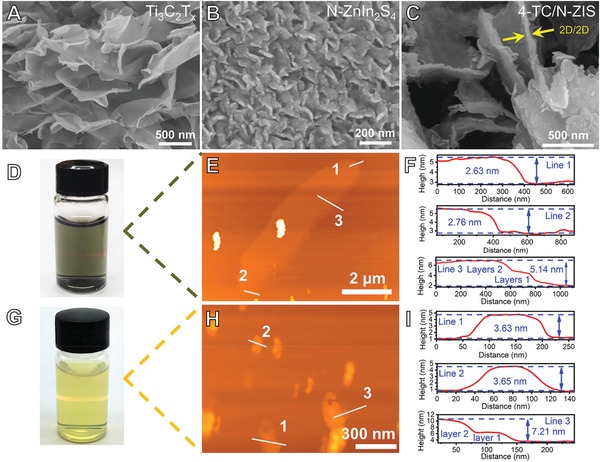
SEM images of A) Ti_3_C_2_T*_x_*, B) N‐ZnIn_2_S_4_, and C) 4‐TC/N‐ZIS composite, the photographs of the Tyndall effect of the D) Ti_3_C_2_T*_x_* and G) N‐ZnIn_2_S_4_ colloidal solution, AFM and height profile of E,F) few‐layered Ti_3_C_2_T*_x_* and H,I) N‐ZnIn_2_S_4_.

The crystal structure of all samples was analyzed by X‐ray diffraction (XRD), as can be seen in the XRD pattern of Ti_3_C_2_T*_x_* (Figure 1B), the main peak for the (104) plane of Ti_3_AlC_2_ disappeared, indicating that Al was removed from the Ti_3_AlC_2_ and formed the Ti_3_C_2_T*_x_* MXene. Moreover, the peaks located at 7.7° (002), 16.6° (004), and 60.7° (110) further demonstrated the successful formation of the Ti_3_C_2_T*_x_* MXene.^[^ ^33^, ^37^ ^]^ Figure 1C shows that the diffraction peaks at 22.3°, 27.5°, 30.5°, 47.3°, 52.5°, and 55.7° were well consistent with the (006), (102), (104), (110), (116), and (022) planes of ZnIn_2_S_4_, indicating that the F‐ZnIn_2_S_4_, L‐ZnIn_2_S_4_, and N‐ZnIn_2_S_4_ were successfully prepared and in the hexagonal phase structures (JCPDS No. 65–2023).^[^ ^38^ ^]^ It was worth noting that the peaks of F‐ZnIn_2_S_4_ were sharper than those of L‐ZnIn_2_S_4_ and N‐ZnIn_2_S_4_. It can be obviously observed from the SEM images of F‐ZnIn_2_S_4_ (Figure S2A, Supporting Information), L‐ZnIn_2_S_4_ (Figure S2B, Supporting Information), and N‐ZnIn_2_S_4_ (**Figure** **2**B) that the F‐ZnIn_2_S_4_ shows the shape of large flower‐like particle while the L‐ZnIn_2_S_4_ was made up of the layer‐by‐layer stacked small flakes. Therefore, the sharper XRD peaks of F‐ZnIn_2_S_4_ may be attributed to the larger crystalline grain size of F‐ZnIn_2_S_4_ than that of the L‐ZnIn_2_S_4_ and N‐ZnIn_2_S_4_.^[^ ^39^ ^]^ Figure 1D shows the XRD pattern of a series of *x*‐TC/N‐ZIS composites, which can be observed that the diffraction peaks of the *x*‐TC/N‐ZIS composites were similar to those of N‐ZnIn_2_S_4_. However, the diffraction peaks at (002) shifted to the lower degree relative to N‐ZnIn_2_S_4_, which can be ascribed to the increased layer spacing of N‐ZnIn_2_S_4_ and the dispersion of N‐ZnIn_2_S_4_ attached to the Ti_3_C_2_T*_x_* surface.^[^ ^40^ ^]^ Due to the low content of Ti_3_C_2_T*_x_* and its high dispersion, the diffraction peaks of the Ti_3_C_2_T*_x_* cannot be observed.

The N_2_ adsorption–desorption isotherms of F‐ZnIn_2_S_4_, L‐ZnIn_2_S_4_, N‐ZnIn_2_S_4_, Ti_3_C_2_T*_x_*, and 4‐TC/N‐ZIS samples were shown in Figure S7A (Supporting Information). It can be seen that the N‐ZnIn_2_S_4_ nanosheets were in line with the H4 hysteresis loop ^[^ ^41^ ^]^, indicating that there were narrow fissure pores between the N‐ZnIn_2_S_4_ nanosheets. Moreover, the Brunauer–Emmett–Teller (BET) specific surface area of N‐ZnIn_2_S_4_ was 123.82 m^2^ g^−1^, which was 1.56 times than that of F‐ZnIn_2_S_4_ and 2.12 times than that of L‐ZnIn_2_S_4_, respectively (Table S1, Supporting Information), indicating the successful exfoliation of L‐ZnIn_2_S_4_. After N‐ZnIn_2_S_4_ was coupled with Ti_3_C_2_T*_x_* to form the TC/N‐ZIS composite, the BET specific surface area was slightly reduced compared with N‐ZnIn_2_S_4_ (Figure S7 and Table S1, Supporting Information), but was slightly increased relative to Ti_3_C_2_T*_x_* powder (48.09 m^2^ g^−1^), which can be attributed to the formation of the compact interface between the Ti_3_C_2_T*_x_* and the N‐ZnIn_2_S_4_.

The morphology and the thickness of the Ti_3_C_2_T*_x_*, N‐ZnIn_2_S_4_, and 4‐TC/N‐ZIS were investigated by scanning electron microscopy (SEM) and atomic force microscopy (AFM) measurements. The SEM images of the Ti_3_C_2_T*_x_* powder showed that the Ti_3_C_2_T*_x_* is in the 2D nanosheet structure, and no agglomeration or restacking phenomena can be observed (Figure S8A, Supporting Information; Figure 2A). In addition, the Ti_3_C_2_T*_x_* showed highly dispersed large flakes and slight wrinkles on the surface and edge, which might be ascribed to the surface energy change of the MXene after inducing NH_4_^+^.^[^ ^42^ ^]^ Figure S8B (Supporting Information) and Figure 2B and show that the N‐ZnIn_2_S_4_ was in a curly and fluffy foam shape, which was composed of multiple small flakes with a size of 200–500 nm. Moreover, there were gaps between these small N‐ZnIn_2_S_4_ flakes, which was beneficial to the redispersion of the N‐ZnIn_2_S_4_ in water. From the AFM images of the Ti_3_C_2_T*_x_* and the N‐ZnIn_2_S_4_, it can be observed that the size of the N‐ZnIn_2_S_4_ nanosheet is smaller than that of the Ti_3_C_2_T*_x_* (Figure 2E,H). The height profiles (Figure 2F,I) corresponding to the white line in the AFM images showed that the thickness of Ti_3_C_2_T*_x_* and N‐ZnIn_2_S_4_ were less than 2.76 and 3.65 nm, respectively, indicating the ultrathin nature of Ti_3_C_2_T*_x_* and N‐ZnIn_2_S_4_ nanosheets.

As shown in Figure 2C, after the integration of the Ti_3_C_2_T*_x_* with the ultrathin N‐ZnIn_2_S_4_ flakes, it can be observed that the N‐ZnIn_2_S_4_ flakes were highly dispersed and immobilized on the 2D few‐layered Ti_3_C_2_T*_x_*, indicating the successful formation of 2D/2D TC/N‐ZIS heterostructure. In addition, the TC/N‐ZIS composite showed the 2D structure, which is beneficial to the charge carriers transfer from the interior of the TC/N‐ZIS photocatalyst to the surface, and initiate the photocatalytic reaction. The energy‐dispersive spectroscopy (EDS) element mapping (Figure S9, Supporting Information) of the 4‐TC/N‐ZIS sample demonstrated the existent of the Zn, In, S, Ti, and C, and these elements were uniformly distributed in the 2D/2D 4‐TC/N‐ZIS composite, which further confirmed the successful coupling of Ti_3_C_2_T*_x_* with N‐ZnIn_2_S_4_. Additionally, the EDS spectrum displayed that the atomic ratio of the Zn, In, and S in the 4‐TC/N‐ZIS heterostructure was 1:1.77:3.19 (Figure S10 and Table S2, Supporting Information), demonstrated the rich S vacancies exist in 4‐TC/N‐ZIS. The SEM images of the other TC/N‐ZIS composites with different content of Ti_3_C_2_T*_x_* can also be found in Figure S11 (Supporting Information).

**Figure 3:**
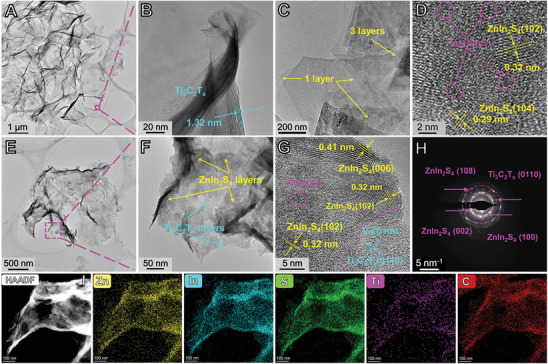
TEM and HRTEM images of A,B) Ti_3_C_2_T*_x_*, C,D) N‐ZnIn_2_S_4_, and E, F, G) 4‐TC/N‐ZIS, H) SAED pattern of 4‐TC/N‐ZIS, I) HAADF‐STEM image and the corresponding EDS element (Zn, In, S, Ti, C) mappings of the 4‐TC/N‐ZIS composite.

The morphology and microstructure of the samples were further investigated by high resolution transmission electron microscopy (HRTEM), as shown in **Figure** **3**. The clean lamellar distribution of Ti_3_C_2_T*_x_* colloid on the copper grid indicated the successful exfoliation (Figure S12, Supporting Information). As shown in the TEM images of the Ti_3_C_2_T*_x_* (Figure 3A), the ultrathin nature and some wrinkles of the few‐layered Ti_3_C_2_T*_x_* can be observed obviously, which was consistent with the SEM results. Moreover, Figure 3B shows cross‐sections of the edge of the exfoliated Ti_3_C_2_T*_x_* nanosheets, and the space between the Ti_3_C_2_T*_x_* flakes was measured to be 1.32 nm. Figure 3C shows the ultrathin nanosheet structure of N‐ZnIn_2_S_4_, which was consistent with the results of SEM and AFM. However, the F‐ZnIn_2_S_4_ sample displayed the tightly packed marigold morphology, indicating the severe agglomeration of the ZnIn_2_S_4_ nanosheets (Figure S13, Supporting Information). In Figure 3D, the fringe spacing of 0.32 and 0.29 nm corresponded to (102) and (104) planes of hexagonal ZnIn_2_S_4_,^[^ ^43^ ^]^ respectively. In addition, apparent shadow regions in the lattice fringe were clearly observed in the purple circles, indicating the presence of S vacancies in N‐ZnIn_2_S_4_.

**Figure 4:**
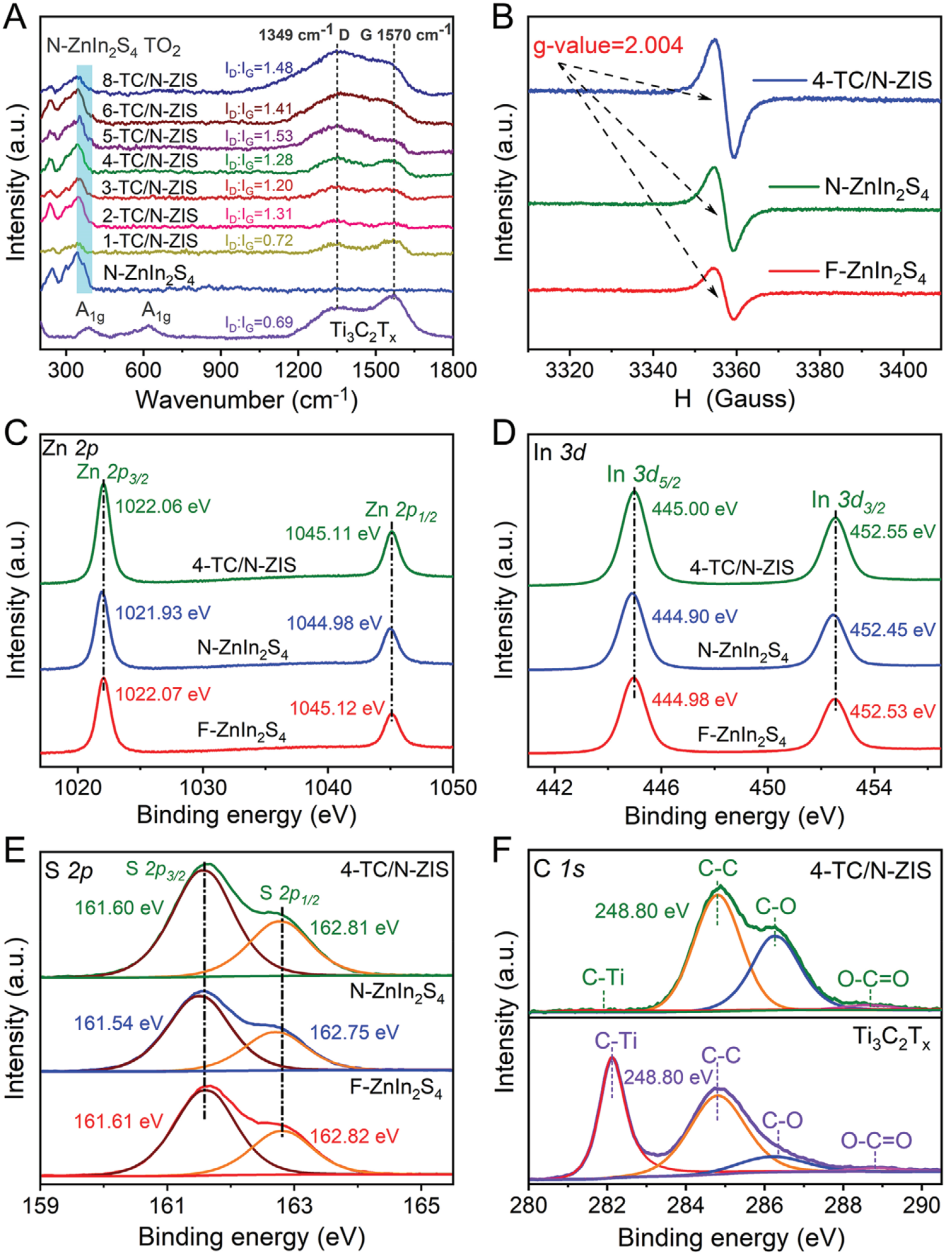
A) Raman spectra of Ti_3_C_2_T*_x_*, N‐ZnIn_2_S_4_, and *x*‐TC/N‐ZIS (*x* = 1, 2, 3,4, 5, 6, and 8), B) ESR spectra F‐ZnIn_2_S_4_, N‐ZnIn_2_S_4_ and 4‐TC/N‐ZIS. High‐resolution XPS peak deconvolution of C) Zn 2p, D) In 3d, and E) S 2p in F‐ZnIn_2_S_4_, N‐ZnIn_2_S_4_, and 4‐TC/N‐ZIS.

The TEM images of the 4‐TC/N‐ZIS composite showed that the ultrathin structure was maintained after the N‐ZnIn_2_S_4_ flakes were immobilized on the surface of the few‐layered Ti_3_C_2_T*_x_* (Figure 3E,F), indicating the successfully constructing of the 2D/2D compact interface between the N‐ZnIn_2_S_4_ and the Ti_3_C_2_T*_x_* nanosheet. As shown in Figure 4G, the lattice fringes spacing of 0.41 and 0.32 nm were in good agreement with the (006) and (102) planes of ZnIn_2_S_4_, respectively, while the lattice fringes spacing of 0.26 nm corresponding to the (0110) plane of Ti_3_C_2_T*_x_*. Moreover, an obvious interface was observed between the N‐ZnIn_2_S_4_ and the Ti_3_C_2_T*_x_* flakes, further demonstrating the existence of the close interfacial contact between these two ultrathin nanosheets. Notably, the vacancies still exist in the 4‐TC/N‐ZIS composite shown in the purple circles in Figure 3G. In Figure 3H, the selected area electron diffraction (SAED) showed the existence of the (002), (108), and (100) plane of ZnIn_2_S_4_ and the (0110) plane of Ti_3_C_2_T*_x_*, respectively, indicating the successful coupling of the ZnIn_2_S_4_ and the Ti_3_C_2_T*_x_* again. In addition, the element mapping corresponding to HAADF‐STEM images displayed that the Zn, In, S, Ti, and C elements were distributed uniformly in the ultrathin 4‐TC/N‐ZIS sample. Based on the above results, it can be concluded that the 2D/2D ZnIn_2_S_4_/Ti_3_C_2_T*_x_* heterostructure was successfully formed.

### 2.2 Surface Chemical State, Vacancy, and Electronic Structure

Raman spectra, ESR spectra, and XPS were carried out to investigate the surface chemical states, vacancies, and electronic structures of the photocatalysts. The Raman spectra of the F‐ZnIn_2_S_4_ and N‐ZnIn_2_S_4_ (Figure S14, Supporting Information) showed peaks at 247.4, 297.4, and 344.2 cm^−1^, corresponding to the longitudinal optical mode (LO_1_), longitudinal optical mode (LO_2_), and transverse optical mode (TO_2_) of ZnIn_2_S_4_, respectively.^[^ ^13^, ^44^ ^]^ From the Raman spectra of Ti_3_C_2_T*_x_*, the peaks at 387.1 and 622.4 cm^−1^ corresponded to the A_1g_ signal of the –OH, –O, and –F groups on the surface of Ti_3_C_2_T*_x_* (**Figure** **4**A), and the absence of Ti‐Al vibration further indicated the success of etching.^[^ ^45^ ^]^ Notably, the obvious signals at 1349 and 1470 cm^−1^ corresponded to D and G bands of graphitized carbon generated during the etching process, which was consistent with the previous report.^[^ ^37^ ^]^ Interestingly, the graphitized carbon can act as the electron trap to capture photogenerated electrons, which is also beneficial to the separation of electron and holes.^[^ ^32^, ^46^ ^]^ Moreover, the derivative graphitized carbon is closely connected to the Ti_3_C_2_T*_x_*, therefore, the carbon species seems to function as an electron channel between the Ti_3_C_2_T*_x_* and N‐ZnIn_2_S_4_ for accelerating the electron migration. The Raman spectra of *x*‐TC/N‐ZIS showed the vibration peaks of Ti_3_C_2_T*_x_* and N‐ZnIn_2_S_4_, indicating the coexistence of Ti_3_C_2_T*_x_* and N‐ZnIn_2_S_4_ in the *x*‐TC/N‐ZIS composite. Compared to N‐ZnIn_2_S_4_, the TO_2_ vibration signals of all *x*‐TC/N‐ZIS redshift to the higher wavenumber. Moreover, the *I*_D_/*I*_G_ value (Table S3, Supporting Information) of all the *x*‐TC/N‐ZIS composite increased compared with that of Ti_3_C_2_T*_x_* which indicated a slight damage to the graphitized carbon during the synthetic process. The above results demonstrated strong interaction and enhanced charge transfer between the Ti_3_C_2_T*_x_* and the N‐ZnIn_2_S_4_.^[^ ^47^ ^]^

The Fourier transform infrared spectroscopy (FT‐IR) (Figure S15A, Supporting Information) showed three peaks at 3401, 1615, and 1396 cm^−1^ for F‐ZnIn_2_S_4_, L‐ZnIn_2_S_4_, and N‐ZnIn_2_S_4_, which corresponding to the absorbed water and hydroxyl groups on the surface. In addition, a peak at 1109 cm^−1^ can be observed, which corresponded to the C‐O bond generated during the hydrothermal process.^[^ ^48^ ^]^ As can be seen in Figure S15B (Supporting Information), the FT‐IR spectra of the *x*‐TC/N‐ZIS showed similar peaks with that of the N‐ZnIn_2_S_4_, indicated that the functional groups were not removed after the coupling of N‐ZnIn_2_S_4_ with Ti_3_C_2_T*_x_*. Notably, the hydroxyl groups retained on the surface endow the *x*‐TC/N‐ZIS composite with excellent hydrophilicity and thus facilitate photocatalytic water splitting.

To shed light on the sulfur vacancy on ZnIn_2_S_4_, the F‐ZnIn_2_S_4_, N‐ZnIn_2_S_4_, and 4‐TC/N‐ZIS were investigated by electron spin‐resonance (ESR) spectroscopy. As shown in Figure 4B, F‐ZnIn_2_S_4_, N‐ZnIn_2_S_4_, and 4‐TC/N‐ZIS all appeared obvious signal at g = 2.004, indicated the existence of S vacancy.^[^ ^49^ ^]^ In general, the enhancement of the ESR signal indicated the existence of more electron capture center.^[^ ^19^ ^]^ Therefore, as the S vacancies acted as the electron‐trapped center, the stronger ESR signal of N‐ZnIn_2_S_4_ may be attributed to more S vacancies in N‐ZnIn_2_S_4_ than that of F‐ZnIn_2_S_4_. More importantly, the 4‐TC/N‐ZIS sample showed stronger signal at *g* = 2.004 than that of N‐ZnIn_2_S_4_. As a consequence, higher signal at *g* = 2.004 indicated more electron capture center was formed in the 4‐TC/N‐ZIS sample. Based on the previous report,^[^ ^13^, ^50^ ^]^ the sulfur vacancy in ZnIn_2_S_4_ was demonstrated as the electron trap to capture the photogenerated electrons, thus prolonging the charge carrier lifetime and enhancing the photocatalytic hydrogen production rate. Moreover, the S vacancy in ZnIn_2_S_4_ acted as an electron trap and enriched electrons at the S vacancy was demonstrated by using ESR and density functional theory (DFT) in the work of Zhang et al. and Wang et al.^[^ ^16^, ^19^ ^]^ Consequently, the formation of S vacancy in ZnIn_2_S_4_ is conducive to the separation of the photoinduced electrons and holes, and thus enhance the photocatalytic H_2_ evolution performance.

X‐ray photoelectron spectroscopy (XPS) was performed to further demonstrate the existence of sulfur vacancies and the interaction of ZnIn_2_S_4_ and Ti_3_C_2_T*_x_* in heterostructures.^[^ ^51^ ^]^ The XPS survey spectra corresponding to the F‐ZnIn_2_S_4_, N‐ZnIn_2_S_4_, Ti_3_C_2_T*_x_*, and 4‐TC/ZIS were shown in Figure S16 (Supporting Information). The binding energy of Zn 2p in F‐ZnIn_2_S_4_ (Figure 4C) was located at 1022.07 and 1045.12 eV, which was attributed to the Zn 2p_3/2_ and Zn 2p_1/2_ of ZnIn_2_S_4_. The high‐resolution XPS spectra of the Zn 2p in N‐ZnIn_2_S_4_ showed that the binding energy of Zn 2p_3/2_ and Zn 2p_1/2_ negatively shifted to 1021.93 and 1044.98 eV compared to that of F‐ZnIn_2_S_4_. Similarly, the binding energy of In 3d_5/2_ (444.90 eV) and In 3d_3/2_ (452.45 eV) in N‐ZnIn_2_S_4_ was also negatively shifted compared with that of In 3d_5/2_ (444.98 eV) and In 3d_3/2_ (452.53 eV) in F‐ZnIn_2_S_4_ (Figure 4D).^[^ ^52^ ^]^ In addition, the S 2p binding energy of S 2p_3/2_ (161.54 eV) and S 2p_1/2_ (162.75 eV) in N‐ZnIn_2_S_4_ negatively shifted compared to that of S 2p_3/2_ (161.61 eV) and S 2p_1/2_ (162.82 eV) of F‐ZnIn_2_S_4_ (Figure 4E). The negative shift of the binding energy of Zn 2p, In 3d, and S 2p can be attributed to the formation of S vacancies, which resulted in the reduction of coordination number of sulfur atoms and the decrease of the electron cloud density around them.^[^ ^13^, ^19^ ^]^

Interestingly, after 2D/2D contact with Ti_3_C_2_T*_x_* the binding energy of Zn 2p_3/2_ (1022.06 eV), Zn 2p_1/2_ (1045.11 eV), In 3d_5/2_ (445.00 eV), In 3d_3/2_ (452.55 eV), S 2p_3/2_ (161.60 eV), and S 2p_1/2_ (162.81 eV) positively shifted to the higher value than that of N‐ZnIn_2_S_4_ (Figure 4C‐E), indicated the decreased density of outer electrons, which demonstrated that the electrons transferred from the N‐ZnIn_2_S_4_ to Ti_3_C_2_T*_x_*.^[^ ^53^ ^]^

The high‐resolution XPS spectra of C 1s (Figure 4F) showed the binding energy at 282.12, 284.80, 286.35, and 288.81 eV, which corresponding to the C—Ti, C—C, C—O, and O—C═O in Ti_3_C_2_T*_x_*, respectively.^[^ ^54^ ^]^ Compared to the high‐resolution XPS spectra of C in Ti_3_C_2_T*_x_*, the binding energy of C 1s for C—Ti and C—O in 4‐TC/N‐ZIS negatively shifted to 282.01 and 286.24 eV, respectively, further demonstrating the electrons transferred from the N‐ZnIn_2_S_4_ to Ti_3_C_2_T*_x_* through the 2D/2D Ti_3_C_2_T*_x_*/ZnIn_2_S_4_ compact interface. As shown in the high‐resolution XPS spectra of Ti 2p (Figure S17, Supporting Information), the binding energy at 455.13 (461.85), 456.09 (462.04), 457.64 (463.27), and 459.16 eV (464.39 eV) correspond to the Ti–C, Ti^2+^, Ti^3+^, and Ti–O in Ti_3_C_2_T*_x_*, respectively.^[^ ^29^, ^53^ ^]^ However, due to the small amount of Ti_3_C_2_T*_x_*, only weak Ti 2p peaks can be observed in the high‐resolution XPS spectra of Ti 2p in 4‐TC/N‐ZIS (Figure S17, Supporting Information). In the process of assembly, N‐ZnIn_2_S_4_ nanosheet with small size will cover the surface of Ti_3_C_2_T*_x_*, which further leading to the weak signal of the Ti. In order to obtain stronger Ti signal, the XPS spectra was carried out after the 4‐TC/ZIS sample was etched by Ar ion at different of 5, 10, and 15 nm (Figure S18, Supporting Information). However, the results showed that the Ti signal is still low due to the low content of the Ti_3_C_2_T*_x_*.

### 2.3 Band Structure and Photocatalytic Hydrogen Evolution Performance

**Figure 5:**
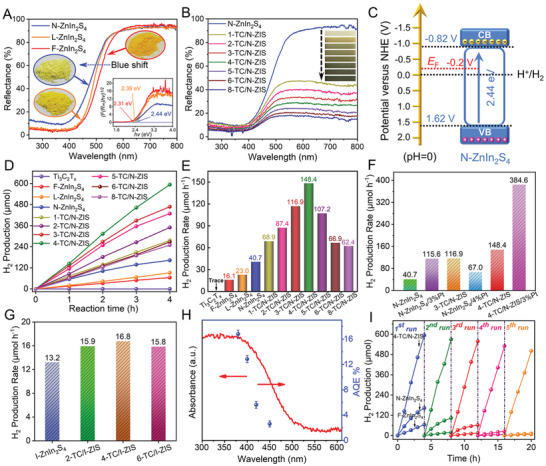
Ultraviolet–visible diffuse reflectance spectra of A) F‐ZnIn_2_S_4_, L‐ZnIn_2_S_4_, N‐ZnIn_2_S_4_ (the insets are the digital photographs of F‐ZnIn_2_S_4_, L‐ZnIn_2_S_4_, and N‐ZnIn_2_S_4_, and the corresponding band gap values calculated by the Kubelka–Munk method), and B) *x*‐TC/N‐ZIS (*x* = 1, 2, 3, 4, 5, 6, and 8, the insets are the colors of the samples with different Ti_3_C_2_T*_x_* content), C) energy band alignment (vs NHE, pH = 0) of N‐ZnIn_2_S_4_, D) time course of photocatalytic H_2_ production performance, E) the photocatalytic H_2_ production rate of Ti_3_C_2_T*_x_*, F‐ZnIn_2_S_4_, L‐ZnIn_2_S_4_, N‐ZnIn_2_S_4_, and *x*‐TC/N‐ZIS samples, F) photocatalytic H_2_ production rate over N‐ZnIn_2_S_4_, N‐ZnIn_2_S_4_/3%Pt, 3‐TC/N‐ZIS, N‐ZnIn_2_S_4_/4%Pt, 4‐TC/N‐ZIS, and 4‐TC/N‐ZIS/3%Pt, H) photocatalytic H_2_ production rate over I‐ZnIn_2_S_4_ and *x*‐TC/I‐ZIS (*x* = 2, 4, and 6), G) the apparent quantum efficiency (AQE) of 4‐TC/N‐ZIS at different wavelength, and I) cyclic experiments of H_2_ production over F‐ZnIn_2_S_4_, N‐ZnIn_2_S_4_, and 4‐TC/N‐ZIS.

The light‐harvesting capability of the photocatalyst was investigated by UV–vis diffuse reflectance spectrum (UV–vis DRS). As shown in **Figure** **5**A, F‐ZnIn_2_S_4_, L‐ZnIn_2_S_4_, and N‐ZnIn_2_S_4_ show obvious absorption of visible light, and the intrinsic absorption edge of the L‐ZnIn_2_S_4_ displayed a clear blue shift compared with that of F‐ZnIn_2_S_4_. Moreover, after the L‐ZnIn_2_S_4_ was exfoliated to ultrathin N‐ZnIn_2_S_4_, the intrinsic absorption edge was further shifted to lower wavelength, which was due to the well‐known quantum size effect.^[^ ^55^ ^]^ In addition, based on the (*F*(*R*_∞_)*hv*)^1/2^ versus photon‐energy plots (inset in Figure 5A), the band gap of the F‐ZnIn_2_S_4_, L‐ZnIn_2_S_4_, and N‐ZnIn_2_S_4_ was measured to be 2.31, 2.39, and 2.44 eV, respectively. Interestingly, the TC/N‐ZIS composites exhibited enhanced significant light absorption compared to N‐ZnIn_2_S_4_. In addition, with the increase of Ti_3_C_2_T*_x_* from 1 to 8 wt%, the light absorption intensity of the TC/N‐ZIS composites gradually increased in the region of 250–800 nm, which was attributed to full‐spectrum absorption of dark Ti_3_C_2_T*_x_* (Figure S19, Supporting Information). In order to accurately obtain the band structure of N‐ZnIn_2_S_4_, the energy of the valance band position relative to Fermi level of N‐ZnIn_2_S_4_ was measured to 1.82 eV by the ultraviolet photoelectron spectrometer (UPS) (Figure S20, Supporting Information). The spectrum was calibrated using Ag standard samples (Figure S21, Supporting Information). Moreover, due to the Fermi level of the semiconductor close to the flat band potential, we obtained Mott–Schottky plot at the frequency of 0.5, 1.0, and 1.5 kHz (Figure S22, Supporting Information) and the value of the flat band potential was converted by Equation (1)^[^ ^56^ ^]^

(1) $$E\left(\mathrm{NHE}\right)=E\left(\mathrm{Ag}/\mathrm{AgCl}\right)+E^{\theta }+0.059\;\mathrm{pH}$$

where *E^*θ*^* (Ag/AgCl) = 0.197 V. The flat‐band potentials of F‐ZnIn_2_S_4_, L‐ZnIn_2_S_4_ and N‐ZnIn_2_S_4_ were calculated to be −0.15, −0.21, and −0.2 V versus NHE (pH = 0), respectively, which met the reduction potential requirement for reducing H^+^ to hydrogen. Besides, the positive value of the slope of the Mott–Schottky plot represents that the three semiconductors were the n‐type semiconductor.^[^ ^57^ ^]^ Thus, combined with the above measured results, the band structure of N‐ZnIn_2_S_4_ can be calculated and summarized in Figure 5C, and the CB, VB, and Fermi level positions of N‐ZnIn_2_S_4_ are located at −0.82, 1.62, and −0.2 V versus NHE (pH = 0), respectively.

The photocatalytic hydrogen production performance of the photocatalyst was investigated under visible light (*λ* ≥ 400 nm) irradiation with triethanolamine (TEOA) as the hole scavenging agent. As shown in Figure 5D,E, the hydrogen production rate of N‐ZnIn_2_S_4_ (40.7 µmol h^−1^) was 1.8 times and 2.5 times higher than that of L‐ZnIn_2_S_4_ (23.0 µmol h^−1^) and F‐ZnIn_2_S_4_ (16.1 µmol h^−1^), respectively, due to the ultrathin 2D structure of the N‐ZnIn_2_S_4_ and the existence of S vacancy on the surface. The hydrogen production rate of the TC/N‐ZIS composites was improved with Ti_3_C_2_T*_x_* as the cocatalyst, and the 4‐TC/N‐ZIS sample exhibited the optimal hydrogen production rate of 148.4 µmol h^−1^, which is 3.6 times and 9.2 times higher than that of N‐ZnIn_2_S_4_ and F‐ZnIn_2_S_4_, respectively. However, when the content of Ti_3_C_2_T*_x_* was increased and higher than 4 wt%, the hydrogen production rate of TC/N‐ZIS composites decreased gradually, which can be attributed to the coverage of active sites on the N‐ZnIn_2_S_4_ caused by excessive Ti_3_C_2_T*_x_*. In order to investigate the effect of specific surface area on the photocatalytic hydrogen evolution performance, we calculated the hydrogen production rate per surface area of the samples (Table S4, Supporting Information). It can be observed that the hydrogen production rate per surface area of the 4‐TC/N‐ZIS sample reached 71.17 µmol h^−1^ m^−2^, which is 4.3 times and 7.1 times higher than that of the N‐ZnIn_2_S_4_ (16.48 µmol h^−1^ m^−2^) and F‐ZnIn_2_S_4_ (10.06 µmol h^−1^ m^−2^). The results further demonstrated that the enhanced photocatalytic hydrogen evolution performance was attributed to the fast interfacial charge transfer at the compact 2D/2D heterointerfaces between the ZnIn_2_S_4_ and Ti_3_C_2_T*_x_*, and the sulfur vacancy on the N‐ZnIn_2_S_4_ further enhanced the separation of the photoinduced electrons and holes.

As a contrast, F‐ZnIn_2_S_4_ was coupled with Ti_3_C_2_T*_x_* to form the 4‐TC/F‐ZIS, it can be observed from the SEM images of 4‐TC/F‐ZIS that 2D Ti_3_C_2_T*_x_* covered on the surface of F‐ZnIn_2_S_4_ flower sphere (Figure S23, Supporting Information). However, most of the F‐ZnIn_2_S_4_ surface was not contacted with the 2D Ti_3_C_2_T*_x_* due to the spherical shape F‐ZnIn_2_S_4_, and the F‐ZnIn_2_S_4_ can only vertically contacted with the 2D Ti_3_C_2_T*_x_* instead of the formation of 2D/2D interface. In this case, the hydrogen evolution rate (39.2 µmol h^−1^) of 4‐TC/F‐ZIS showed only 2.4 times higher than that (16.0 µmol h^−1^) of F‐ZnIn_2_S_4_ (Figure S24, Supporting Information). These results indicated that the construction of 2D/2D interface was crucial to shortening the electron transport path between ZnIn_2_S_4_ and Ti_3_C_2_T*_x_* and enhanced the separation efficiency of photogenerated electrons and holes. To compare the Ti_3_C_2_T*_x_* with noble metal as the cocatalyst, N‐ZnIn_2_S_4_ was also loaded with Pt metal and their photocatalytic hydrogen evolution performance was investigated. After the loading with 3% Pt (N‐ZnIn_2_S_4_/3%Pt), the photocatalytic hydrogen evolution rate (115.6 µmol h^−1^) of N‐ZnIn_2_S_4_/3%Pt was 2.8 times higher than that of N‐ZnIn_2_S_4_ (40.7 µmol h^−1^). And the 3‐TC/N‐ZIS showed comparative hydrogen evolution rate (116.9 µmol h^−1^) to that of N‐ZnIn_2_S_4_/3%Pt. Notably, the hydrogen production rate of 4‐TC/N‐ZIS was 2.2 times higher than that of the N‐ZnIn_2_S_4_/4%Pt, which demonstrated that 2D Ti_3_C_2_T*_x_* was an efficient cocatalyst for promoting photocatalytic hydrogen production (Figure 5F). Interestingly, after loading with 3% Pt on 4‐TC/N‐ZIS, the hydrogen production rate reached 384.6 µmol h^−1^ on account of the synergistic effect between Ti_3_C_2_T*_x_* and Pt.

In addition, the *x*‐TC/I‐ZIS composites were also prepared by in situ growth of ZnIn_2_S_4_ on the Ti_3_C_2_T*_x_* surface and used for the photocatalytic hydrogen evolution. From the XRD pattern (Figure S25, Supporting Information) and the SEM images (Figure S26, Supporting Information) of the *x*‐TC/I‐ZIS composites, it can be observed that the *x*‐TC/I‐ZIS was successfully prepared and the surface of the Ti_3_C_2_T*_x_* surface was covered by ZnIn_2_S_4_. As shown in Figure 8D, the hydrogen evolution rate of 2‐TC/I‐ZIS, 4‐TC/I‐ZIS, and 6‐TC/I‐ZIS was only 1.2, 1.3, and 1.2 times higher than that of I‐ZIS (13.2 µmol h^−1^), which might be ascribed to the reason that the most active sites on the Ti_3_C_2_T*_x_* surface were covered by ZnIn_2_S_4_, leading to the decreased photocatalytic hydrogen evolution rate compared to that of 4‐TC/N‐ZIS. The apparent quantum efficiency (AQE) of 4‐TC/N‐ZIS at different wavelengths was investigated, and their relationship with the optical absorption properties of 4‐TC/N‐ZIS was shown in Figure 8E. The AQE of the 4‐TC/N‐ZIS at 380, 400, 420, and 450 nm were 16.75%, 12.84%, 5.62%, and 2.61%, respectively. In addition, the AQE of the N‐ZnIn_2_S_4_/3%Pt at 400 nm reached 28.61%. Notably, the AQE of 4‐TC/N‐ZIS was in direct proportion to the light absorption intensity as shown in Figure 8H, indicating that the absorption capacity of photons is one of the important factors determining hydrogen production efficiency. It is worth mentioning that the 4‐TC/N‐ZIS composite represents better photocatalytic hydrogen production performance compared with some reported ZnIn_2_S_4_‐based photocatalysts under visible light (Table S5, Supporting Information).

The stability test was carried out to evaluate the cycling stability of the F‐ZnIn_2_S_4_, N‐ZnIn_2_S_4_, and 4‐TC/N‐ZIS, the results were shown in Figure 8I. Obviously, the hydrogen production of F‐ZnIn_2_S_4_ and N‐ZnIn_2_S_4_ decreased by 96.4% and 98.1%, respectively after five cycles. To explore the reasons for the instability of the N‐ZnIn_2_S_4_, the N‐ZnIn_2_S_4_ sample after reaction was analyzed by XPS. From the high‐resolution XPS spectrum of N‐ZnIn_2_S_4_, the valence states of Zn and In did not change, indicating that they were not reduced during the reaction. However, in addition to S^2−^, the element S appeared in the elemental phase of S^0^ (Figure S27, Supporting Information), which indicated that the photooxidation corrosion of N‐ZnIn_2_S_4_ occurred during the reaction.^[^ ^58^ ^]^ Notably, the hydrogen production of the 4‐TC/N‐ZIS sample decreased only by 15.6% after 5 cycles, indicating the enhanced stability of the 4‐TC/N‐ZIS. XRD pattern and Raman spectra displayed that the crystal structure of the 4‐TC/N‐ZIS did not change before and after reaction (Figure S28, Supporting Information). The high‐resolution XPS spectrum of the used 4‐TC/N‐ZIS sample showed that the chemical composition of the 4‐TC/N‐ZIS composites did not change after reaction (Figure S29, Supporting Information). In addition, the SEM image (Figure S30, Supporting Information) showed that the 4‐TC/N‐ZIS still possessed the nanosheet structure, and the contact interface between N‐ZnIn_2_S_4_ and Ti_3_C_2_T*_x_* can still be observed from the HRTEM image (Figure S31, Supporting Information), indicating the high stability of the 2D/2D TC/N‐ZIS heterointerface. Moreover, according to the previous research,^[^ ^31^ ^]^ the absorption effect of Ti_3_C_2_T*_x_* on dissolved ions might restrain the photocorrosion reaction of ZnIn_2_S_4_. The above results indicated that the main reasons for the decrease of the activity of the 4‐TC/N‐ZIS sample may be as follows. First, during the photocatalytic reaction process, the agglomeration of photocatalyst is inevitable, which will occlude the active sites of the photocatalysts. Second, the mechanical force caused by the stirring can give rise to the slight microstructure collapse of 4‐TC/N‐ZIS, which is harmful to stability of the 2D/2D Ti_3_C_2_T*_x_*/ZnIn_2_S_4_ interface and the separation of the photogenerated electrons and holes. Thirdly, although a great deal of the photogenerated holes generated by ZnIn_2_S_4_ in the reaction process were consumed by the sacrificial agent, there may still some holes react with S^2−^ to form the S^0^, leading to partial decomposition of the ZnIn_2_S_4_. Therefore, the decrease of photocatalytic activity might be due to combined result of the above‐mentioned factors.

### 2.4 Charge Separation and Transfer

**Figure 6:**
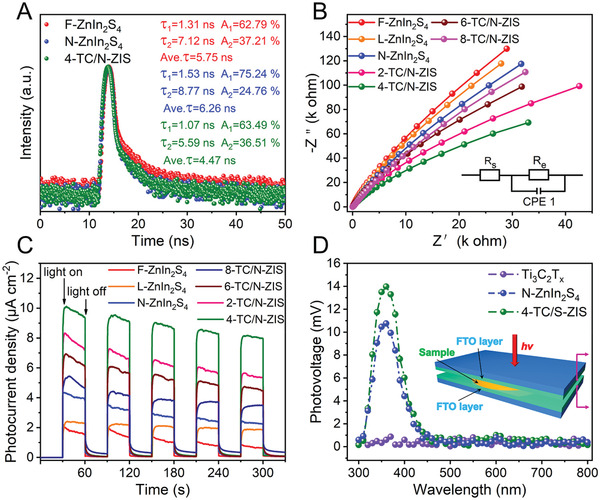
A) Time‐resolved transient photoluminescence (TRPL) decay spectra of F‐ZnIn_2_S_4_, N‐ZnIn_2_S_4_, and 4‐TC/N‐ZIS, B) EIS Nyquist plots and C) transient photocurrent responses of F‐ZnIn_2_S_4_, L‐ZnIn_2_S_4_, N‐ZnIn_2_S_4_, and *x*‐TC/N‐ZIS (*x* = 2, 4, 6, 8), and D) surface photovoltage spectra (SPV) of Ti_3_C_2_T*_x_*, N‐ZnIn_2_S_4_, and 4‐TC/N‐ZIS.

To further reveal the synergistic effect of sulfur vacancy and Ti_3_C_2_T*_x_* on the separation efficiency of electron–hole pairs on N‐ZnIn_2_S_4_, photoelectrochemical measurement were carried out. From the solid state photoluminescence (PL) spectra in Figure S32A (Supporting Information), compared with F‐ZnIn_2_S_4_, the significant decreased fluorescence signal at about 476 nm of ultrathin N‐ZnIn_2_S_4_ indicated that the recombination of photogenerated electrons and holes was greatly restrained.^[^ ^59^ ^]^ More importantly, the fluorescence peak signals of TC/N‐ZIS were further decreased compared to that of N‐ZnIn_2_S_4_ (Figure S32B, Supporting Information), demonstrating the positive effect of Ti_3_C_2_T*_x_* for effectively suppress the charge carrier recombination. In order to further understand the electron transfer mechanism in the ZnIn_2_S_4_ and TC/N‐ZIS composites, the carrier dynamics of the photocatalysts were further detected by time‐resolved fluorescence decay spectroscopy (TRPL) (**Figure** **6**A), and the average fluorescence lifetime (*τ*_A_) was calculated by Equation (2)^[^ ^12^, ^60^ ^]^

(2) $$\tau_{A}=\frac{A_{1}{\tau_{1}}^{2}+A_{2}{\tau_{2}}^{2}}{A_{1}\tau_{1}+A_{2}\tau_{2}}$$

where *τ*_1_ is generated by the nonradiative recombination of charge carriers in the defect states of ZnIn_2_S_4_, and *τ*_2_ is caused by the recombination of free excitons,^[^ ^12^ ^]^ *A*_1_ and *A*_2_ correspond to the amplitudes respectively. The calculated average lifetime (*τ*_A_ = 6.26 ns) of the N‐ZnIn_2_S_4_ was longer than that of F‐ZnIn_2_S_4_ (*τ*_A_ = 5.75 ns) indicated that the S vacancy can reduce the photogenerated charge carriers recombination by capturing electrons and serving as their host.^[^ ^13b^ ^]^ Of course, compared with F‐ZnIn_2_S_4_, the ultrathin structure of N‐ZnIn_2_S_4_ shortens the carrier transmission distance and reduces its recombination rate in the migration process. Notably, the calculated average lifetime of 4‐TC/N‐ZIS (*τ*_A_ = 4.47 ns) was shorter than that of N‐ZnIn_2_S_4_, which was due to reason that the photoinduced electrons on N‐ZnIn_2_S_4_ could rapidly transfer to the Ti_3_C_2_T*_x_* via the 2D/2D interface. In addition, the 4‐TC/N‐ZIS composites displayed the shortest lifetime relative to 2‐TC/N‐ZIS, 6‐TC/N‐ZIS, and 8‐TC/N‐ZIS (Figure S33, Supporting Information). The above results of charge carrier migration dynamics indicated that the synergistic effect of the Ti_3_C_2_T*_x_* and S vacancy greatly improved the separation of the photogenerated electrons and holes, which is beneficial to enhance the photocatalytic hydrogen production performance.^[^ ^19^ ^]^

In addition, from the EIS Nyquist plots shown in Figure 6B, the arc radius of *x*‐TC/N‐ZIS (*x* = 2, 4, 6, and 8) was smaller than those of N‐ZnIn_2_S_4_, F‐ZnIn_2_S_4_, and L‐ZnIn_2_S_4_, indicating the better charge transfer efficiency of the *x*‐TC/N‐ZIS due to the 2D/2D interface between the few‐layered Ti_3_C_2_T*_x_* and N‐ZnIn_2_S_4_. Notably, the minimal arc radius of Ti_3_C_2_T*_x_* was attributed to its excellent electrical conductivity, which is conducive to the electron transport (Figure S34, Supporting Information). Moreover, It should be noted that a higher photogenerated current suggests a more efficient separation of photogenerated electron–hole pairs. Higher photocurrent density as shown in Figure 6C indicates again N‐ZnIn_2_S_4_ has better photogenerated carrier separation ability than F‐ZnIn_2_S_4_. The 4‐TC/N‐ZIS possessed the highest photocurrent density, further demonstrating the efficient charge carrier transfer and separation on the 4‐TC/N‐ZIS composite. Surface photovoltage measurement (SPV) measurement was carried out to investigate the surface charge carrier transfer on the 4‐TC/N‐ZIS composite.^[^ ^61^ ^]^

As shown in Figure 6D, an obvious positive signal of N‐ZnIn_2_S_4_ in the range of 300–450 nm can be observed, indicated that the N‐ZnIn_2_S_4_ was a n‐type semiconductor, which was consistent with the results of Mott–Schottky.^[^ ^62^ ^]^ No signal could be detected for the Ti_3_C_2_T*_x_* sample, indicated that the metallic Ti_3_C_2_T*_x_* did not exhibit photoexcitation property. Compared with N‐ZnIn_2_S_4_ (10.8 mV), the stronger peak signal (14.1 mV) of 4‐TC/N‐ZIS indicating that more photogenerated electrons were transferred to the 4‐TC/N‐ZIS surface, thus forming a stronger surface‐to‐bulk electric field based on favorable electron transfer capability of Ti_3_C_2_T*_x_*. The above results of photoelectrochemical characterizations confirmed the excellent separation of electron–hole pairs on the 4‐TC/N‐ZIS composite, which is beneficial to the photocatalytic hydrogen evolution from water.

### 2.5 Theoretical Calculation

**Figure 7:**
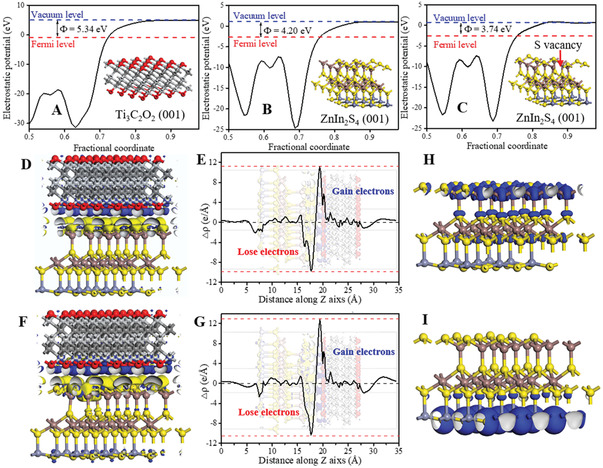
Electrostatic potentials of the A) Ti_3_C_2_O_2_, B) ZnIn_2_S_4_, and the C) ZnIn_2_S_4_ with S vacancy. Charge density difference and planar‐averaged electron density difference along with Z direction of Ti_3_C_2_O_2_/ZnIn_2_S_4_ heterojunction D,E) without and F,G) with the S vacancy (blue and yellow areas indicate the loss and accumulation of electrons, respectively). H) Distribution of partial charge density near the edge of the conduction band and I) valence band for ZnIn_2_S_4_.

DFT calculations were used to reveal the charge migration on the 2D/2D Ti_3_C_2_T*_x_*/N‐ZnIn_2_S_4_ heterojunction interface. Due to the O‐terminated Ti_3_C_2_T*_x_* with a low content of –OH and –F terminal groups can be obtained by the LiF/HCl etching method,^[^ ^63^ ^]^ to get closer to reality, we reckon without –OH and –F terminal groups and used the Ti_3_C_2_O_2_ slab for the DFT calculation. As shown in **Figure** **7**A‐C, the work functions of Ti_3_C_2_O_2_, ZnIn_2_S_4_, and ZnIn_2_S_4_ with S vacancy were calculated to be 5.34, 4.20, and 3.74 eV, respectively, which indicates that the electrons on the ZnIn_2_S_4_ and ZnIn_2_S_4_ with S vacancy can transfer to the Ti_3_C_2_O_2_ due to the higher work function of the Ti_3_C_2_O_2_. The charge carriers transfer pathway of the Ti_3_C_2_O_2_/ZnIn_2_S_4_ interfaces was demonstrated by the result of the electron density difference as depicted in Figure 7D. Strikingly, the electrons on the ZnIn_2_S_4_ were transferred to the Ti_3_C_2_O_2_, corroborating the strong electron coupling effect between the Ti_3_C_2_O_2_ and ZnIn_2_S_4_. Figure 7E plots the planar‐averaged charge density difference along the Z direction to directly display the change of charge density. Evidently, the electrons mainly transfer from the ZnIn_2_S_4_ to Ti_3_C_2_O_2_, which is consistent with the XPS results.

Moreover, after a S vacancy was removed on the ZnIn_2_S_4_ surface, more electrons can transfer from the ZnIn_2_S_4_ to Ti_3_C_2_O_2_ (Figure 7F,G), indicated that the surface S vacancy on the ZnIn_2_S_4_ surface can further facilitate the electron migration and enhance the separation of the photogenerated electrons and holes. Notably, according to the distribution of partial charge density near the edge of the conduction band (Figure 7H) and valence band (Figure 7I) of ZnIn_2_S_4_, the conduction band minimum (CBM) was located at the In–S atomic layer, and the valence band maximum (VBM) was located at the Zn–S atomic layer. For the 2D/2D Ti_3_C_2_O_2_/ZnIn_2_S_4_ heterojunction, the Ti_3_C_2_O_2_ surface is in contact with the In‐S terminated surface of ZnIn_2_S_4_, i.e., the Ti_3_C_2_O_2_ is close to the CBM of the ZnIn_2_S_4_, which further accelerated the photogenerated electron transfer from ZnIn_2_S_4_ to Ti_3_C_2_O_2_ and greatly benefited the separation of the photogenerated electrons and holes.

### 2.6 Possible Photocatalytic Mechanism

**Figure 8:**
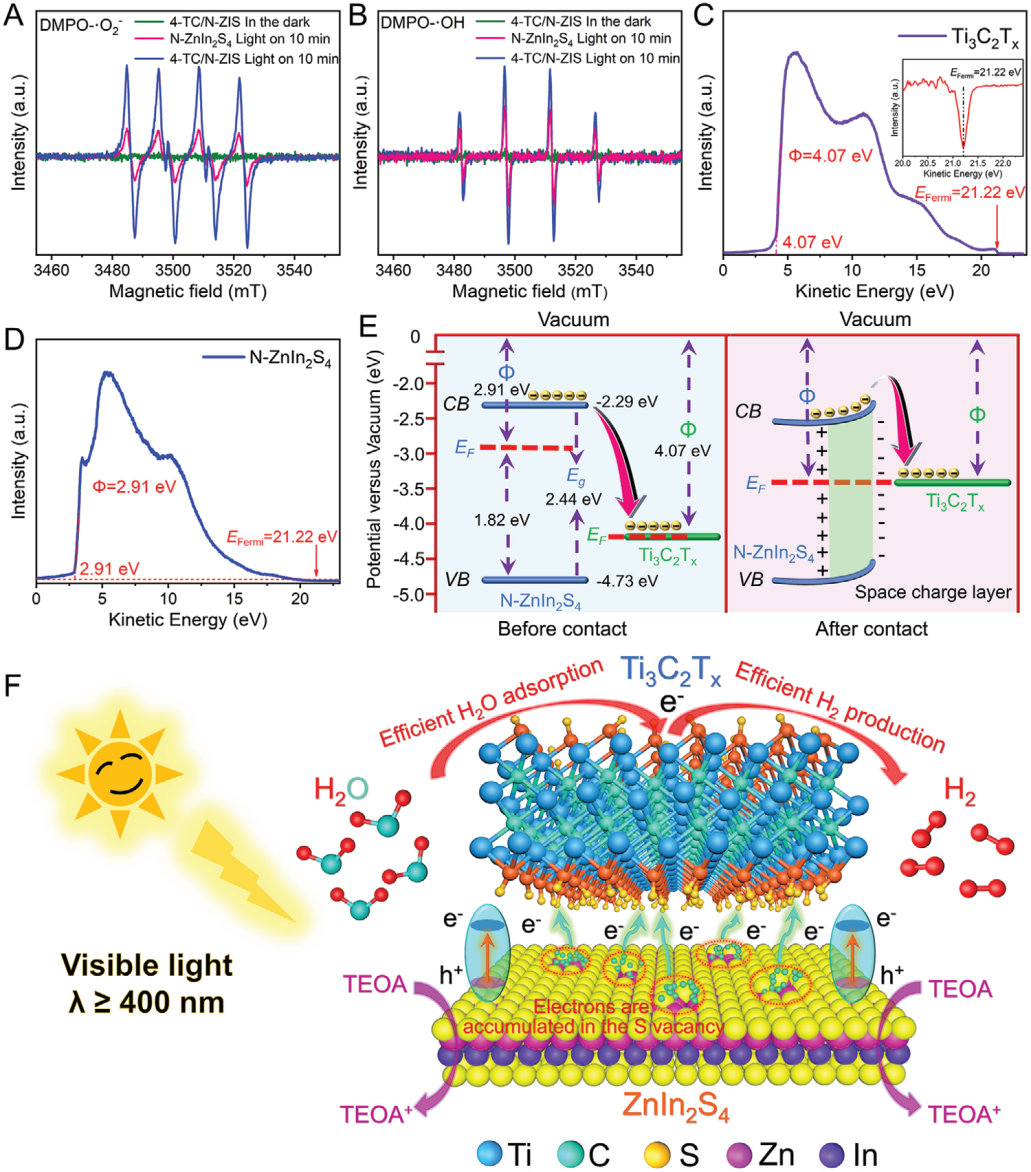
EPR spectra of radical adducts signal labeled by DMPO for A) ⋅O^2−^ and B) ⋅OH of N‐ZnIn_2_S_4_ and 4‐TC/N‐ZIS, UPS spectrum of C) Ti_3_C_2_T*_x_* (the inset showed the integral model of secondary electron cutoff after correction) and D) N‐ZnIn_2_S_4_ and E) energy scheme before and after the contact of Ti_3_C_2_T*_x_* and N‐ZnIn_2_S_4_. F) Schematic illustration of photocatalytic hydrogen production mechanism in 4‐TC/N‐ZIS.

Electron paramagnetic resonance (EPR) can effectively capture free radicals to determine the active groups produced during the photocatalytic reaction.^[^ ^43a^ ^]^ Superoxide radical (·O^2−^) and hydroxyl radical (·OH) signals were detected by using DMPO as the spin marker in aqueous and methanol solutions, respectively. **Figure** **8**A,B displays that there were no ·O^2−^ and ·OH signals for both N‐ZnIn_2_S_4_ and 4‐TC/N‐ZIS in the dark. After 10 min irradiation under the Xenon lamp with a 400 nm cutoff filter, the 4‐TC/N‐ZIS sample showed a stronger quadruplex ·O^2−^ peak signal than that of N‐ZnIn_2_S_4_ with the intensity ratio of 1:2:2:1, indicating more ·O^2−^ can be generated in the 4‐TC/N‐ZIS composite. Similarly, a higher quadruplex ·OH peak than that of N‐ZnIn_2_S_4_ with the intensity ratio of 1:1:1:1 indicated that more ·OH can be excited in the 4‐TC/N‐ZIS composite under the visible light irradiation. Since superoxide (·O^2−^) and hydroxyl (·OH) radicals are produced by holes and photogenerated electrons, respectively, ^[^ ^32^ ^]^ the higher signal of 4‐TC/N‐ZIS indicated superior separation of charge carriers. As a consequence, the 4‐TC/N‐ZIS composite exhibited better photocatalytic hydrogen evolution performance than that of the N‐ZnIn_2_S_4_.

In addition, in order to further understand the electron transfer behavior between the Ti_3_C_2_T*_x_* and N‐ZnIn_2_S_4_ in the TC/N‐ZIS composites, the energy level structures of Ti_3_C_2_T*_x_* and N‐ZnIn_2_S_4_ were discussed based on the vacuum energy level. UPS was used to determine the electronic structures of Ti_3_C_2_T*_x_* and N‐ZnIn_2_S_4_ and calibrated by Ag standard sample, and a bias voltage of −5 eV was used for all measurements (Figure S35, Supporting Information). The work function (*Φ*) of the photocatalyst was calculated according to Equation (3) and (4)^[^ ^64^ ^]^

(3) Φ=hv+W

(4) $$W=E_{\mathrm{cutoff}}-E_{\mathrm{Fermi}}$$

where *hv*, *W*, *E*_cutoff_, and *E*_Fermi_ are the incident photon energy, the width of the UPS spectrum, the kinetic energy of cutoff edge, and Fermi edge, respectively.

As shown in Figure 8C,D, the work functions of N‐ZnIn_2_S_4_ and Ti_3_C_2_T*_x_* were calculated to be 2.91 and 4.07 eV, respectively. Therefore, the positions of the corresponding Fermi energy level of the N‐ZnIn_2_S_4_ and Ti_3_C_2_T*_x_* relative to the vacuum energy level were −2.91 and −4.07 eV, respectively. It is worth noting that the work functions of N‐ZnIn_2_S_4_ (2.91 eV) and Ti_3_C_2_T*_x_* (4.07 eV) obtained from the UPS spectrum are different from the calculated work functions of ZnIn_2_S_4_ with S vacancy (3.74 eV) and Ti_3_C_2_O_2_ (5.34 eV) based on the DFT. The discrepancy can be ascribed to the complex surface structures of the N‐ZnIn_2_S_4_ and Ti_3_C_2_T*_x_*. For example, there are some groups, such as –OH, –F, –O, on the surface of Ti_3_C_2_T*_x_*. For simplicity, we used ZnIn_2_S_4_ (001) and Ti_3_C_2_O_2_ (001) for the DFT calculation, which can also help us to better understand the nature of the material. However, for the sake of rigor, we used the experimental value obtained from the UPS spectrum as the standard to determine the position of energy level.

As a result, under light irradiation, the electrons excited on N‐ZnIn_2_S_4_ will transfer to Ti_3_C_2_T*_x_* when the 2D/2D interface was formed between the N‐ZnIn_2_S_4_ and Ti_3_C_2_T*_x_*, which was consistent with the results of XPS. According to the previous UPS valence band spectrum of N‐ZnIn_2_S_4_ (Figure S20, Supporting Information), the position of VB (*E*_VB_ vs Fermi level) of N‐ZnIn_2_S_4_ was −1.82 eV, which was −4.73 eV relative to the vacuum level. Since the band gap value (*E*_g_) of N‐ZnIn_2_S_4_ was 2.44 eV based on the UV–vis DRS, and according to the equation of *E*_CB_ = *E*_VB_ − *E*_g_,^[^ ^53^ ^]^ the CB position of the N‐ZnIn_2_S_4_ relative to the vacuum energy level was −2.29 eV. In this case, the Fermi energy level (*E*_F_) of N‐ZnIn_2_S_4_ was closer to its CB, which in line with the characteristics of n‐type semiconductor and was consistent with the Mott–Schottky and SPV results. According to the above results, the positions of the energy level of N‐ZnIn_2_S_4_ and Ti_3_C_2_T*_x_* relative to the vacuum level before and after the contact of N‐ZnIn_2_S_4_ with Ti_3_C_2_T*_x_* was shown in Figure 8E. Specifically, after the N‐ZnIn_2_S_4_ was combined with the Ti_3_C_2_T*_x_* to form the 2D/2D Ti_3_C_2_T*_x_*/N‐ZnIn_2_S_4_ interface, the photogenerated electrons generated on the CB of N‐ZnIn_2_S_4_ will transfer to Ti_3_C_2_T*_x_* due to the smaller work function of N‐ZnIn_2_S_4_. Although the contact interface between N‐ZnIn_2_S_4_ and Ti_3_C_2_T*_x_* is only a few hundred nanometers, electrons can still accumulate on the Ti_3_C_2_T*_x_* side and lead to the positive charge of N‐ZnIn_2_S_4_ side. Therefore, a space charge layer at the interface can be formed while this space charge layer may be extremely thin. Notably, the S vacancies inside N‐ZnIn_2_S_4_ can act as efficient electron trap to suppress the recombination of photogenerated electron–hole pairs,^[^ ^13b^ ^]^ which promotes the accumulation of electrons on the surface S vacancies of N‐ZnIn_2_S_4_ nanosheets. Moreover, due to the electron harvesting effect of Ti_3_C_2_T*_x_*, the recombination of the electrons and holes will be greatly reduced. In the meantime, the CB and VB of N‐ZnIn_2_S_4_ near the 2D/2D Ti_3_C_2_T*_x_*/N‐ZnIn_2_S_4_ interface are bent “upward” to form the Schottky junction. Although the existence of potential Schottky barrier will increase the difficulty of electron transfer to a certain extent, the photogenerated electrons on N‐ZnIn_2_S_4_ are able to cross the barrier and transfer to Ti_3_C_2_T*_x_*. Moreover, the formed Schottky barrier could restrain the backflow of electrons from Ti_3_C_2_T*_x_* to N‐ZnIn_2_S_4_ and boost the separation of photogenerated electrons and holes, which will greatly enhance photocatalytic hydrogen evolution performance.

Based on the photocatalytic experiments, characterization analysis, and the DFT calculation, the mechanism of charge transfer and photocatalytic hydrogen production over 2D/2D Ti_3_C_2_T*_x_*/N‐ZnIn_2_S_4_ composites was proposed (Figure 8F). Under light irradiation, the electrons excited from the VB of the N‐ZnIn_2_S_4_ to CB, and the photogenerated electrons migrate to the Ti_3_C_2_T*_x_* spontaneously. The 2D/2D Ti_3_C_2_T*_x_*/N‐ZnIn_2_S_4_ heterointerfaces provide wide and shortest paths for the transfer of charge carriers, thus restraining the recombination of photoinduced electrons and holes. The Ti_3_C_2_T*_x_* serves as the electron accepter and active sites to promote hydrogen evolution from the water. Simultaneously, the photogenerated holes on the VB of the ZnIn_2_S_4_ nanosheet were scavenged by sacrificial agents. Noteworthy, the sulfur vacancies in ZnIn_2_S_4_ act as electron traps further facilitate charge carrier transfer at the 2D/2D interface and enhance the separation of the photoinduced electron–hole pairs, which was verified by the characterization analysis and DFT calculation. Moreover, the formation of the Schottky barrier between the N‐ZnIn_2_S_4_ and Ti_3_C_2_T*_x_* intimate contact interface can inhibit the backflow of electrons from the Ti_3_C_2_T*_x_* to N‐ZnIn_2_S_4_, further suppressing the recombination of the photoinduced electrons and holes.^[^ ^65^ ^]^ In short, benefiting from the synergistic effect of the 2D/2D interface, S vacancy, and Ti_3_C_2_T*_x_* cocatalyst, the separated photoinduced electrons and holes, the photocatalytic hydrogen evolution performance of the N‐ZnIn_2_S_4_ was greatly enhanced.

## 3 Conclusion

In summary, the 2D/2D Ti_3_C_2_T*_x_*/N‐ZnIn_2_S_4_ composites were successfully synthesized by coupling ultrathin N‐ZnIn_2_S_4_ with few‐layered Ti_3_C_2_T*_x_*. The unique 2D/2D compact interface of the Ti_3_C_2_T*_x_*/N‐ZnIn_2_S_4_ composites provided the broad and short electron transfer paths, which significantly enhanced the transfer and separation of photoinduced charge carriers. The S vacancies in the ultrathin N‐ZnIn_2_S_4_ can serve as an electron trap, which facilitate the separation of charge carriers and greatly accelerated electrons transferred from the N‐ZnIn_2_S_4_ surface to Ti_3_C_2_T*_x_*. In addition, the formation of the Schottky barrier at the 2D/2D Ti_3_C_2_T*_x_*/N‐ZnIn_2_S_4_ interface inhibited the backflow of electrons from the Ti_3_C_2_T*_x_* to N‐ZnIn_2_S_4_, further enhanced the separation of the photoinduced electron–hole pairs. Experimental characterization analysis and DFT calculations demonstrated that the rapid transfer and separation of photoexcited charge carriers is attributed to the strong interaction between the 2D/2D Ti_3_C_2_T*_x_*/N‐ZnIn_2_S_4_ intimate interface and the S‐vacancy on the ZnIn_2_S_4_. The optimal 4‐TC/N‐ZIS composite exhibited a high photocatalytic hydrogen evolution rate of 148.4 µmol h^−1^, which is 3.6 times and 9.2 times higher than that of the N‐ZnIn_2_S_4_ and F‐ZnIn_2_S_4_, respectively. This work revealed the intrinsic principle of the enhanced photocatalytic performance of 2D/2D Ti_3_C_2_T*_x_*/N‐ZnIn_2_S_4_ composites and provides a new way for the construction of highly efficient photocatalysts with 2D/2D heterostructure.

## 4 Experimental Section

### 4.1 Synthesis of ZnIn_2_S_4_ Nanoflower

F‐ZnIn_2_S_4_ was synthesized by the hydrothermal method. Specifically, 1 mmol ZnCl_2_ and 2 mmol InCl_3_⋅4H_2_O were dissolved in 60 mL deionized (DI) water by ultrasound. After stirring for 30 min, 8 mmol thioacetamide was added to the solution, and kept stirring for 1 h. The mixed solution was transferred into a 100 mL Teflon‐lined autoclave and heated at 180 °C for 3 h. After naturally cooling to room temperature, yellowish precipitate was collected and washed with DI water and anhydrous ethanol 4 times respectively by centrifugation to obtain a yellow ZnIn_2_S_4_ precipitate. Subsequently, then the nanoflower ZnIn_2_S_4_ powder was obtained by freeze‐drying the yellow precipitate, and was denoted as F‐ZnIn_2_S_4_.

### 4.2 Synthesis of Layered ZnIn_2_S_4_

Typically, 1 mmol ZnCl_2_, 2 mmol InCl_3_·4H_2_O, and 0.9 g trisodium citrate dihydrate was dissolved in 60 mL DI water. After stirring for 30 min, a transparent solution was obtained. The subsequent process was the same as that for the synthesis of F‐ZnIn_2_S_4_ nanoflower, and the obtained layered ZnIn_2_S_4_ was denoted as L‐ZnIn_2_S_4_.

### 4.3 Synthesis of Sulfur Vacancy‐Rich ZnIn_2_S_4_ Nanosheet

Sulfur vacancy‐rich ultrathin ZnIn_2_S_4_ was prepared by exfoliating the layered ZnIn_2_S_4_. Briefly, the washed and undried layered ZnIn_2_S_4_ was redispersed into 150 mL DI water, and exfoliated by ultrasonic treatment for 40 min at 5 °C in a high‐power ultrasonic machine with circulating cooling water. After that, the transparent solution was centrifuged at 7000 rpm to obtain the sulfur vacancy‐rich ultrathin ZnIn_2_S_4_ supernatant. The ZnIn_2_S_4_ colloidal solution with a concentration of 2 mg mL^−1^. The sulfur vacancy‐rich ultrathin ZnIn_2_S_4_ nanosheets were obtained by freeze‐drying, which was denoted as N‐ZnIn_2_S_4_.

### 4.4 Synthesis of Few‐Layered Ti_3_C_2_T*_x_*

Few‐layered Ti_3_C_2_T*_x_* nanosheets were synthesized by a modified reported method.^[^ ^36^ ^]^ Typically, 3.2 g LiF and 40 mL of 9 m HCl was added to a 100 mL Teflon vessel and stirred for 20 min to dissolve the LiF completely. Subsequently, 2.0 g Ti_3_AlC_2_ powder (Foshan XinXi Technology Co. Ltd, 99.5%, 400 mesh) was slowly added into the above mixed solution and stirred at 40 °C for 48 h. After that, a black suspension was obtained and was centrifugally washed with DI water at 8500 rpm multiple times until the pH is greater than 6. Then, the precipitate after washing was dispersed in 200 mL DI water, and was treated by ultrasonic treatment for 2 h in the Ar atmosphere at 5 °C. Whereafter, a black colloidal solution was obtained and centrifuged at 6000 rpm for 30 min, then the few‐layered Ti_3_C_2_T*_x_* colloidal solution with a concentration of 1.5 mg mL^−1^ was collected.

### 4.5 Synthesis of 2D/2D Ti_3_C_2_T*_x_*/N‐ZnIn_2_S_4_

To prepare the 2D/2D Ti_3_C_2_T*_x_*/N‐ZnIn_2_S_4_ with different amounts of the Ti_3_C_2_T*_x_*, different volumes of few‐layered Ti_3_C_2_T*_x_* colloidal solution (1.5 mg mL^−1^) were added to 50 mL of N‐ZnIn_2_S_4_ colloid (2.0 mg mL^−1^) and sonicated at 5 °C in the Ar atmosphere for 5 min. Then, 10 mL of 1 m NH_4_HCO_3_ solution was gradually added to the above mixed solution under stirring conditions, and kept stirring for 2 h and sonicating in the Ar atmosphere for 10 min. After standing at 5 °C for 1 h, the flocculent precipitate can be formed and sunk to the bottom of the beaker. After separating the supernatant, the flocculent precipitate was dried by vacuum freeze drying to obtain the 2D/2D Ti_3_C_2_T*_x_*/N‐ZnIn_2_S_4_ powder. Finally, the 2D/2D Ti_3_C_2_T*_x_*/N‐ZnIn_2_S_4_ powder was annealed in a tube furnace at 180 °C under Ar atmosphere for 6 h. The 2D/2D Ti_3_C_2_T*_x_*/N‐ZnIn_2_S_4_ with different amounts (1, 2, 3, 4, 5, 6, and 8 wt%) of Ti_3_C_2_T*_x_* was denoted as 1‐TC/N‐ZIS, 2‐TC/N‐ZIS, 3‐TC/N‐ZIS, 4‐TC/N‐ZIS, 5‐TC/N‐ZIS, 6‐TC/N‐ZIS, and 8‐TC/N‐ZIS, respectively. The Ti_3_C_2_T*_x_* powder was prepared by the same method without adding the N‐ZnIn_2_S_4_.

For comparison, the 2D/2D Ti_3_C_2_T*_x_*/N‐ZnIn_2_S_4_ photodeposition with Pt and the Ti_3_C_2_T*_x_*/ZnIn_2_S_4_ composites with different Ti_3_C_2_T*_x_* content obtained by in situ growth of ZnIn_2_S_4_ on the Ti_3_C_2_T*_x_* was also prepared, and these samples were denoted as *x*‐TC/I‐ZIS (*x* = 2, 4, and 8 wt%), the experimental details were shown in the Supporting Information.

### 4.6 Photocatalytic Hydrogen Evolution

The photocatalytic water splitting performance of the photocatalyst was tested in a 275 mL quartz reactor. Typically, 20 mg photocatalyst was dispersed in a mixed solution containing 10 mL TEOA and 40 mL DI water by ultrasonic treatment for 30 min. Before illumination, the photocatalytic reaction system was degassed with ultrahigh pure argon gas for 30 min. The light source was a 300 W Xenon lamp (CEL‐HXF300, Beijing China Education Au‐light Co., Ltd.) equipped with a 400 nm cutoff filter. In addition, the suspension solution was stirred during the photocatalytic hydrogen evolution reaction, and the temperature of the reactor was kept at 25 °C by circulating cooling water. The hydrogen was quantitatively detected by a gas chromatography (GC‐2018, SHIMADZU) equipped with a 5 Å molecular sieve column, a TCD detector, and with Ar as the carrier gas. The experimental details and the calculation method for the apparent quantum efficiency (AQE) are supplemented in the Supporting Information.

### 4.7 Characterization

The characterization methods and DFT calculation are supplemented in the Supporting Information.

## Conflict of Interest

The authors declare no conflict of interest.

## Supporting information

Supporting Information

## Data Availability

The data that support the findings of this study are available from the corresponding authors upon reasonable request.

## References

[1] a) A. Fujishima , K. Honda , Nature 1972, 238, 37; 12635268 10.1038/238037a0

[2] a) Q. Wang , K. Domen , Chem. Rev. 2020, 120, 919; 31393702 10.1021/acs.chemrev.9b00201

[3] Y. Li , C. Zhang , T.‐T. Zhuang , Y. Lin , J. Tian , X.‐Y. Qi , X. Li , R. Wang , L. Wu , G.‐Q. Liu , T. Ma , Z. He , H.‐B. Sun , F. Fan , H. Zhu , S.‐H. Yu , J. Am. Chem. Soc. 2021, 143, 7013. 33929193 10.1021/jacs.1c01514

[4] L. Lin , Z. Lin , J. Zhang , X. Cai , W. Lin , Z. Yu , X. Wang , Nat. Catal. 2020, 3, 649.

[5] A. Nakada , D. Kato , R. Nelson , H. Takahira , M. Yabuuchi , M. Higashi , H. Suzuki , M. Kirsanova , N. Kakudou , C. Tassel , T. Yamamoto , C. M. Brown , R. Dronskowski , A. Saeki , A. Abakumov , H. Kageyama , R. Abe , J. Am. Chem. Soc. 2021, 143, 2491. 33417448 10.1021/jacs.0c10288

[6] H. Lin , Z. Ma , J. Zhao , Y. Liu , J. Chen , J. Wang , K. Wu , H. Jia , X. Zhang , X. Cao , X. Wang , X. Fu , J. Long , Angew. Chem., Int. Ed. 2021, 60, 1235. 10.1002/anie.202009267 33026673

[7] a) L. Sun , Y. Zhuang , Y. Yuan , W. Zhan , X.‐J. Wang , X. Han , Y. Zhao , Adv. Energy Mater. 2019, 9, 1902839;

[8] T. Su , Q. Shao , Z. Qin , Z. Guo , Z. Wu , ACS Catal. 2018, 8, 2253.

[9] a) B. Lin , A. Chaturvedi , J. Di , L. You , C. Lai , R. Duan , J. Zhou , B. Xu , Z. Chen , P. Song , J. Peng , B. Ma , H. Liu , P. Meng , G. Yang , H. Zhang , Z. Liu , F. Liu , Nano Energy 2020, 76, 104972;

[10] Z. Lei , W. You , M. Liu , G. Zhou , T. Takata , M. Hara , K. Domen , C. Li , Chem. Commun. 2003, 17, 2142. 10.1039/b306813g 13678171

[11] B. Liu , X. Liu , J. Liu , C. Feng , Z. Li , C. Li , Y. Gong , L. Pan , S. Xu , C. Q. Sun , Appl. Catal., B 2018, 226, 234.

[12] M.‐Q. Yang , Y.‐J. Xu , W. Lu , K. Zeng , H. Zhu , Q.‐H. Xu , G. W. Ho , Nat. Commun. 2017, 8, 14224. 28146147 10.1038/ncomms14224 PMC5296640

[13] a) X. Jiao , Z. Chen , X. Li , Y. Sun , S. Gao , W. Yan , C. Wang , Q. Zhang , Y. Lin , Y. Luo , Y. Xie , J. Am. Chem. Soc. 2017, 139, 7586; 28514178 10.1021/jacs.7b02290

[14] X. Zhang , Q. Liao , Z. Kang , B. Liu , X. Liu , Y. Ou , J. Xiao , J. Du , Y. Liu , L. Gao , L. Gu , M. Hong , H. Yu , Z. Zhang , X. Duan , Y. Zhang , Adv. Mater. 2021, 33, 2007051. 10.1002/adma.202007051 33448081

[15] D. Ray , B. Voigt , M. Manno , C. Leighton , E. S. Aydil , L. Gagliardi , Chem. Mater. 2020, 32, 4820.

[16] Y. Wang , D. Chen , L. Qin , J. Liang , Y. Huang , Phys. Chem. Chem. Phys. 2019, 21, 25484. 31714570 10.1039/c9cp04709c

[17] Y. Cao , L. Guo , M. Dan , D. E. Doronkin , C. Han , Z. Rao , Y. Liu , J. Meng , Z. Huang , K. Zheng , P. Chen , F. Dong , Y. Zhou , Nat. Commun. 2021, 12, 1675. 33723264 10.1038/s41467-021-21925-7 PMC7960986

[18] F. Gao , R. Lei , X. Huang , J. Yuan , C. Jiang , W. Feng , L. Zhang , P. Liu , Appl. Catal., B 2021, 292, 120187.

[19] S. Zhang , X. Liu , C. Liu , S. Luo , L. Wang , T. Cai , Y. Zeng , J. Yuan , W. Dong , Y. Pei , Y. Liu , ACS Nano 2018, 12, 751. 29261276 10.1021/acsnano.7b07974

[20] M. Naguib , M. Kurtoglu , V. Presser , J. Lu , J. Niu , M. Heon , L. Hultman , Y. Gogotsi , M. W. Barsoum , Adv. Mater. 2011, 23, 4248. 21861270 10.1002/adma.201102306

[21] C. Zhang , B. Anasori , A. Seral‐Ascaso , S.‐H. Park , N. McEvoy , A. Shmeliov , G. S. Duesberg , J. N. Coleman , Y. Gogotsi , V. Nicolosi , Adv. Mater. 2017, 29, 1702678. 10.1002/adma.201702678 28741695

[22] W. Zhao , Y. Lei , Y. Zhu , Q. Wang , F. Zhang , X. Dong , H. N. Alshareef , Nano Energy 2021, 86, 106120.

[23] Z. Wu , C. Li , Z. Li , K. Feng , M. Cai , D. Zhang , S. Wang , M. Chu , C. Zhang , J. Shen , Z. Huang , Y. Xiao , G. A. Ozin , X. Zhang , L. He , ACS Nano 2021, 15, 5696. 33624496 10.1021/acsnano.1c00990

[24] A. Iqbal , F. Shahzad , K. Hantanasirisakul , M.‐K. Kim , J. Kwon , J. Hong , H. Kim , D. Kim , Y. Gogotsi , C. M. Koo , Science 2020, 369, 446. 32703878 10.1126/science.aba7977

[25] D. Pinto , B. Anasori , H. Avireddy , C. E. Shuck , K. Hantanasirisakul , G. Deysher , J. R. Morante , W. Porzio , H. N. Alshareef , Y. Gogotsi , J. Mater. Chem. A 2020, 8, 8957.

[26] Q. Zhang , H. Lai , R. Fan , P. Ji , X. Fu , H. Li , ACS Nano 2021, 15, 5249. 33617227 10.1021/acsnano.0c10671

[27] Y. Li , H. Shao , Z. Lin , J. Lu , L. Liu , B. Duployer , P. O. Å. Persson , P. Eklund , L. Hultman , M. Li , K. Chen , X.‐H. Zha , S. Du , P. Rozier , Z. Chai , E. Raymundo‐Piñero , P.‐L. Taberna , P. Simon , Q. Huang , Nat. Mater. 2020, 19, 894. 32284597 10.1038/s41563-020-0657-0

[28] Y. Liu , H. Xiao , W. A. Goddard , J. Am. Chem. Soc. 2016, 138, 15853. 27960324 10.1021/jacs.6b10834

[29] T. Su , Z. D. Hood , M. Naguib , L. Bai , S. Luo , C. M. Rouleau , I. N. Ivanov , H. Ji , Z. Qin , Z. Wu , Nanoscale 2019, 11, 8138. 30788480 10.1039/c9nr00168a

[30] J. Ran , G. Gao , F.‐T. Li , T.‐Y. Ma , A. Du , S.‐Z. Qiao , Nat. Commun. 2017, 8, 13907. 28045015 10.1038/ncomms13907 PMC5512649

[31] X. Xie , N. Zhang , Z.‐R. Tang , M. Anpo , Y.‐J. Xu , Appl. Catal., B 2018, 237, 43.

[32] G. Zuo , Y. Wang , W. L. Teo , A. Xie , Y. Guo , Y. Dai , W. Zhou , D. Jana , Q. Xian , W. Dong , Y. Zhao , Angew. Chem., Int. Ed. 2020, 59, 11287. 10.1002/anie.202002136 32250502

[33] A. Lipatov , M. Alhabeb , M. R. Lukatskaya , A. Boson , Y. Gogotsi , A. Sinitskii , Adv. Electron. Mater. 2016, 2, 1600255.

[34] Y. He , H. Rao , K. Song , J. Li , Y. Yu , Y. Lou , C. Li , Y. Han , Z. Shi , S. Feng , Adv. Funct. Mater. 2019, 29, 1905153.

[35] S. Mypati , A. Sellathurai , M. Kontopoulou , A. Docoslis , D. P. J. Barz , Carbon 2021, 174, 581.

[36] J. Peng , X. Chen , W.‐J. Ong , X. Zhao , N. Li , Chem 2019, 5, 18.

[37] Z. Li , W. Huang , J. Liu , K. Lv , Q. Li , ACS Catal. 2021, 11, 8510.

[38] G. Zhang , D. Chen , N. Li , Q. Xu , H. Li , J. He , J. Lu , Angew. Chem., Int. Ed. 2020, 59, 8255. 10.1002/anie.202000503 31989737

[39] B. Luo , Y. Fang , B. Wang , J. Zhou , H. Song , L. Zhi , Energy Environ. Sci. 2012, 5, 5226.

[40] X. Wu , B. Huang , Q. Wang , Y. Wang , Chem. Eng. J. 2020, 380, 122456.

[41] W. Guan , Y. Zhang , Y. Chen , J. Wu , Y. Cao , Y. Wei , P. Huo , J. Catal. 2021, 396, 40.

[42] S. Zhang , P. Huang , J. Wang , Z. Zhuang , Z. Zhang , W.‐Q. Han , J. Phys. Chem. Lett. 2020, 11, 1247. 31994884 10.1021/acs.jpclett.9b03682

[43] a) J. Hu , C. Chen , Y. Zheng , G. Zhang , C. Guo , C. M. Li , Small 2020, 16, 2002988; 10.1002/smll.202002988 32776442

[44] Y. Chen , S. Hu , W. Liu , X. Chen , L. Wu , X. Wang , P. Liu , Z. Li , Dalton Trans. 2011, 40, 2607. 21290033 10.1039/c0dt01435d

[45] A. Sarycheva , Y. Gogotsi , Chem. Mater. 2020, 32, 3480.

[46] B. Wang , M. Wang , F. Liu , Q. Zhang , S. Yao , X. Liu , F. Huang , Angew. Chem., Int. Ed. 2020, 59, 1914. 10.1002/anie.201913095 31710145

[47] a) P. Pachfule , D. Shinde , M. Majumder , Q. Xu , Nat. Chem. 2016, 8, 718; 27325100 10.1038/nchem.2515

[48] B. Gao , L. Liu , J. Liu , F. Yang , Appl. Catal., B 2013, 129, 89.

[49] a) Y. Yin , J. Han , Y. Zhang , X. Zhang , P. Xu , Q. Yuan , L. Samad , X. Wang , Y. Wang , Z. Zhang , P. Zhang , X. Cao , B. Song , S. Jin , J. Am. Chem. Soc. 2016, 138, 7965; 27269185 10.1021/jacs.6b03714

[50] Y. Qin , H. Li , J. Lu , Y. Feng , F. Meng , C. Ma , Y. Yan , M. Meng , Appl. Catal., B 2020, 277, 119254.

[51] C. Lv , L. Zhong , Y. Yao , D. Liu , Y. Kong , X. Jin , Z. Fang , W. Xu , C. Yan , K. N. Dinh , M. Shao , L. Song , G. Chen , S. Li , Q. Yan , G. Yu , Chem 2020, 6, 2690.

[52] C. Lv , L. Zhong , H. Liu , Z. Fang , C. Yan , M. Chen , Y. Kong , C. Lee , D. Liu , S. Li , J. Liu , L. Song , G. Chen , Q. Yan , G. Yu , Nat. Sustain. 2021, 4, 868.

[53] S. Cao , B. Shen , T. Tong , J. Fu , J. Yu , Adv. Funct. Mater. 2018, 28, 1800136.

[54] a) C. Yang , Q. Jiang , W. Li , H. He , L. Yang , Z. Lu , H. Huang , Chem. Mater. 2019, 31, 9277;

[55] Y. Li , M. Yang , Y. Xing , X. Liu , Y. Yang , X. Wang , S. Song , Small 2017, 13, 1701552. 10.1002/smll.201701552 28692776

[56] L. Jiao , D. Zhang , Z. Hao , F. Yu , X.‐J. Lv , ACS Catal. 2021, 11, 8727.

[57] P. Xia , S. Cao , B. Zhu , M. Liu , M. Shi , J. Yu , Y. Zhang , Angew. Chem. 2020, 132, 5256. 10.1002/anie.201916012 31944512

[58] J. Wang , Y. Zhang , X. Wang , W. Su , Appl. Catal., B 2020, 268, 118444.

[59] a) P. Li , Z. Zhou , Q. Wang , M. Guo , S. Chen , J. Low , R. Long , W. Liu , P. Ding , Y. Wu , Y. Xiong , J. Am. Chem. Soc. 2020, 142, 12430; 32530616 10.1021/jacs.0c05097

[60] Q. Zhang , J. Zhang , X. Wang , L. Li , Y.‐F. Li , W.‐L. Dai , ACS Catal. 2021, 11, 6276.

[61] J. Ran , H. Zhang , J. Qu , J. Shan , S. Chen , F. Yang , R. Zheng , J. Cairney , L. Song , L. Jing , S.‐Z. Qiao , ACS Mater. Lett. 2020, 2, 1484.

[62] Y. Liu , Y.‐H. Li , X. Li , Q. Zhang , H. Yu , X. Peng , F. Peng , ACS Nano 2020, 14, 14181. 33012166 10.1021/acsnano.0c07089

[63] M. A. Hope , A. C. Forse , K. J. Griffith , M. R. Lukatskaya , M. Ghidiu , Y. Gogotsi , C. P. Grey , Phys. Chem. Chem. Phys. 2016, 18, 5099. 26818187 10.1039/c6cp00330c

[64] J.‐Y. Li , Y.‐H. Li , F. Zhang , Z.‐R. Tang , Y.‐J. Xu , Appl. Catal., B 2020, 269, 118783.

[65] T. Su , R. Peng , Z. D. Hood , M. Naguib , I. N. Ivanov , J. K. Keum , Z. Qin , Z. Guo , Z. Wu , ChemSusChem 2018, 11, 688. 29281767 10.1002/cssc.201702317

