# Supplementary material for: Sulfur Vacancy and Ti3C2T x Cocatalyst Synergistically Boosting Interfacial Charge Transfer in 2D/2D Ti3C2T x /ZnIn2S4 Heterostructure for Enhanced Photocatalytic Hydrogen Evolution
Source: Adv Sci (Weinh). 2021 Nov 21;9(4):2103715. doi: 10.1002/advs.202103715 (PMC8811818; doi:10.1002/advs.202103715)
Supplement: Supplementary file 1 — Supporting Information [file ADVS-9-2103715-s001.pdf]

ADVANCED SCIENCE
Open Access

Supporting Information

for *Adv. Sci.*, DOI: 10.1002/advs.202103715

Sulfur Vacancy and $Ti_3C_2T_x$ Cocatalyst Synergistically Boosting Interfacial Charge Transfer in 2D/2D $Ti_3C_2T_x/ZnIn_2S_4$ Heterostructure for Enhanced Photocatalytic Hydrogen Evolution

Tongming Su, Chengzheng Men, Liuyun Chen, Bingxian Chu, Xuan Luo, Hongbing Ji, Jianhua Chen, and Zuzeng Qin**

Supporting Information

Sulfur Vacancy and $Ti_3C_2T_x$ Cocatalyst Synergistically Boosting Interfacial Charge Transfer in 2D/2D $Ti_3C_2T_x/ZnIn_2S_4$ Heterostructure for Enhanced Photocatalytic Hydrogen Evolution

Tongming Su[1], Chengzheng Men[1], Liuyun Chen[1], Bingxian Chu[1], Xuan Luo[1], Hongbing Ji[1, 2], Jianhua Chen[3], and Zuzeng Qin[1*]*

1. School of Chemistry and Chemical Engineering, Guangxi University, Nanning 530004, P. R. China
2. Fine Chemical Industry Research Institute, School of Chemistry, Sun Yat-sen University, Guangzhou 510275, P. R. China
3. School of Resources, Environment, and Materials, Guangxi University, Nanning 530004, P. R. China

*Corresponding author:

E-mail: sutm@gxu.edu.cn (T. Su); qinzuzeng@gxu.edu.cn (Z. Qin)

1. Experimental Section

Photodeposition of Pt

Typically, H_2PtCl_6 solution (0.1 mg mL^{-1}) was directly added in an aqueous triethanolamine solution containing a photocatalyst, and the amount of H_2PtCl_6 depending on the mass ratio of Pt to the photocatalyst. The reactor was purged with ultra-pure Ar gas for 30 min. A 300 W Xenon lamp (CEL-HXF300, Beijing China Education Au-light Co., Ltd.) equipped with a 400 nm cutoff filter was used as the light source, and Pt species were reduced under light irradiation for 30 min while stirring constantly. Subsequently, the photocatalytic hydrogen evolution reaction was carried out after the reactor was purged with high purity Ar gas for 30 min.

Synthesis of x-TC/I-ZIS by in situ growth method

Different qualities of $Ti_3C_2T_x$ (4.20 mg, 8.46 mg, 16.90 mg) were homodispersed into an aqueous solution of glycerin (50 mL, 20%, pH = 2.5). Then, 1.0 mmol $InCl_3$ •$4H_2O$, 0.5 mmol $ZnCl_2$ and 3.0 mmol thioacetamide were added into the above solution under stirring. The reaction mixture was then stirred in an oil bath at 80 °C for 2 h. After cooling to room temperature, the samples were collected by centrifugation and washed with deionized water and ethanol for 3 times respectively, and the photocatalyst powder was obtained by freeze drying. The synthesized composites were labeled as x-TC/I-ZIS (x = 2 wt.%, 4 wt.%, 8 wt.%), and the sample without $Ti_3C_2T_x$ was labeled as I-$ZnIn_2S_4$.

Apparent quantum efficiency (AQE) measurement

The apparent quantum efficiency was measured in a quartz reactor. The light source was

a 300 W Xenon lamp equipped with the monochromatic filter in different wavelengths of 380 nm, 400 nm, 420 nm, and 450 nm. The light intensity was measured by a CE-NP2000 optical power meter. The AQE can be calculated by Equation:

$$AQE = \frac{2 \times the\ number\ of\ evulved\ H_2\ Molecules}{the\ number\ of\ incident\ photons} \times 100\% \\ = \frac{2 \times r \times N_A \times h \times c}{S \times I \times t \times \lambda} \times 100\% \qquad (S1)$$

Where r is the yield of hydrogen, N_A is Avogadro constant, h is Planck constant, c is the speed of light, S is the illumination area, I is the light intensity, t is the illumination time, and λ is the wavelength of the light source.

2. Characterization

The zeta potential value of the sample was measured on a Nano-ZS90X at 25 °C. Tapping-mode AFM measurement was performed on a 5100N (HITACHI, Japan) atomic force microscope. SEM images were recorded on a field emission scanning electron microscope (HITACHI SU8220, 10 kV). HRTEM images were recorded on a FEI Talos F200X high resolution transmission electron microscope with an acceleration voltage of 300 kV. The X-ray diffraction (XRD) patterns were collected on a SMARTLAB3KW powder diffractometer equipped with a Cu Kα radiation source. Raman spectra were collected on a HORIBA Scientific XploRA Plus spectrometer with laser excitation at 633 nm. Fourier transform infrared (FT-IR) spectra was obtained on a BRUKER TENSOR II infrared spectrometer. The N_2 adsorption and desorption curve, the Brunauer-Emmett-Teller (BET) specific surface area and pore size of the samples were obtained on a TriStar II system. X-ray

photoelectron spectroscopy (XPS) was performed on a Thermo Scientific K-Alpha spectrometer with Al Kα radiation excitation source. Ultraviolet photoelectron spectroscopy (UPS) spectra were collected on a Thermo Fisher Nexsa spectrometer and calibrated with Ag standards. The steady-state fluorescence spectra were recorded using a Thermo Scientific Lumina fluorescence spectrometer. Time-resolved fluorescence spectra was carried out on an Edinburgh FLS 1000 spectrometer equipped with a laser (λ = 365 nm) at room temperature. The UV-Vis diffuse reflectance spectra (UV-Vis DRS) was collected on a TU-19 UV-Vis spectrophotometer. Surface photovoltage spectra (SPV) were recorded on an CEL-SPS 1000 system (Beijing China Education Au-light Co., Ltd.).

Photoelectrochemical measurements

Photoelectrochemical measurements were performed on a CHI760E electrochemical workstation using a three-electrode system with an Ag/AgCl electrode as reference electrode, Pt mesh as the counter electrode, and the photocatalyst as the working electrode. A 0.5 M Na_2SO_4 solution was used as the electrolyte. The working electrodes were prepared as follows: 20 mg catalyst and 20 μL Nafion® solutions were added into 400 μL anhydrous ethanol, and then treated by ultrasonic wave for 90 min, the obtained suspension was uniformly coated on an FTO substrate (1 cm × 1 cm) and dried at room temperature to obtain the working electrode. The transient photocurrent response (TPC) was measured with a 300 W Xenon lamp equipped with a 400 nm cut-off filter as the light source, and the light source was 15 cm away from the electrode surface and the bias voltage was 0.2 V. Electrochemical impedance spectroscopy (EIS) was measured in the dark with an alternating amplitude of 5 mV and a

frequency range of 0.01 kHz to 1000 kHz. The Mott-Schottky curves were measured under the same conditions with an amplitude of 10 mV and frequency of 0.5 kHz, 1 kHz, and 1.5 kHz.

S vacancy capture and Electron paramagnetic resonance (EPR)

All measurements were performed on the Bruker EMX Plus spectrometer. At 77 K, S vacancy capture was performed in the dark. For $\cdot O^{2-}$, 5 mg of catalyst was added to 1 mL methanol, then 45 μL DMPO was added to the above suspension solution, and sonicated for 10 min. For ·OH, 5 mg catalyst was added to 1 mL ultrapure water, followed by adding of 45 μL DMPO and ultrasound for 10 min. A 500 W Xenon lamp with a 400 nm cutoff filter was used as the light source for free radical capture measurement. The first data point was collected in the dark, and the signal was collected at 10 min of illumination.

3. Density function theory calculation details

Cambridge Sequential Total Energy Package (CASTEP) was used to perform all the density functional theory (DFT) calculations within the spin-polarized generalized gradient approximation (GGA) with Perdew–Burke–Ernzerhof (PBE) exchange-correlation functional[1]. The plane-wave energy cutoff was set to 400 eV, and gamma point grid was employed for the Brillouin zone sampling. The convergence criterion of energy and force calculations were set to 10^{-5} eV/atom and 0.03 eV $Å^{-1}$, respectively. The surface computations were performed by using the slab model. The $Ti_3C_2O_2$ (001) and $ZnIn_2S_4$ (001) slabs were built to calculate their work functions, and the work function (E_{wf}) can be calculated by the equation of $E_{wf} = E_{vacuum} - E_{Fermi}$, where the E_{vacuum} and E_{Fermi} are the vacuum energy and

fermi level energy, respectively. For $Ti_3C_2O_2$ (001) slab model, a 4×4×1 supercell with 112 atoms is used for the calculation. For the $ZnIn_2S_4$ (001) slab, a 3×3×1 supercell with 63 atoms was used. A vacuum of approximately 15 Å was placed above the slabs to eliminate the interaction between periodic images. The $ZnIn_2S_4$ (001) slab model, in which a S atom was removed on the slab surface, was used for simulating the $ZnIn_2S_4$ with S vacancy. The $Ti_3C_2T_x/ZnIn_2S_4$ heterostructure model was constructed to investigate the charge transfer between the $Ti_3C_2T_x$ and the $ZnIn_2S_4$. To construct the $Ti_3C_2T_x/ZnIn_2S_4$ interface, a 4×4×1 supercell of $Ti_3C_2O_2$ layer was deposited on the 3×3×1 supercell of $ZnIn_2S_4$ (001) slab, and the lattice mismatch of the interface model was less than 5%. To further investigate the positive effect of the S vacancy for the charge transfer, the $Ti_3C_2O_2/ZnIn_2S_4$ interface with a S atom vacancy on the $ZnIn_2S_4$ (001) slab was also constructed to study the interfacial electron transfer between them. The van der Waals correction was included by using the DFT-D approach.

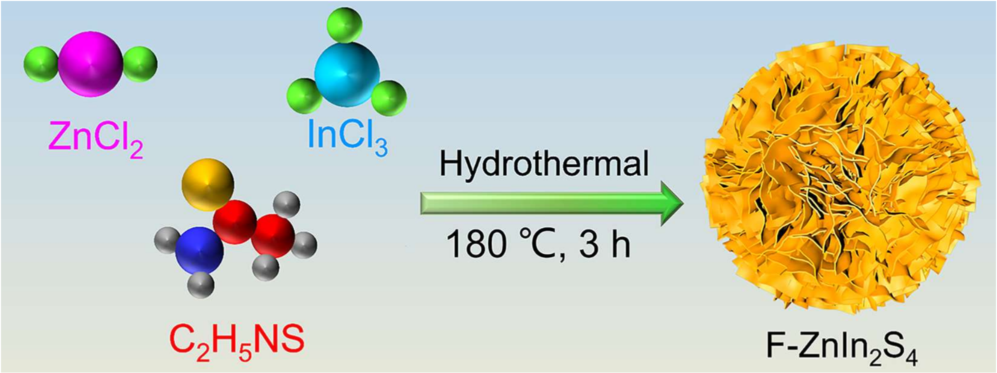

Figure S1. Schematic illustration of the synthetic route of F-$ZnIn_2S_4$.

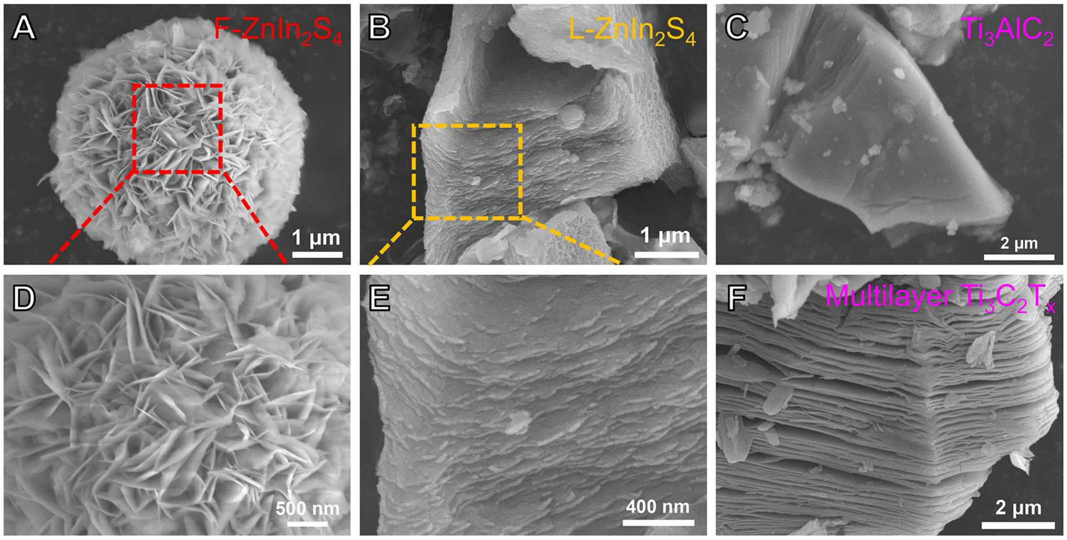

Figure S2. SEM images of (A, D) F-$ZnIn_2S_4$, (B, E) L-$ZnIn_2S_4$, (C) Ti_3AlC_2 and (F) multilayer $Ti_3C_2T_x$.

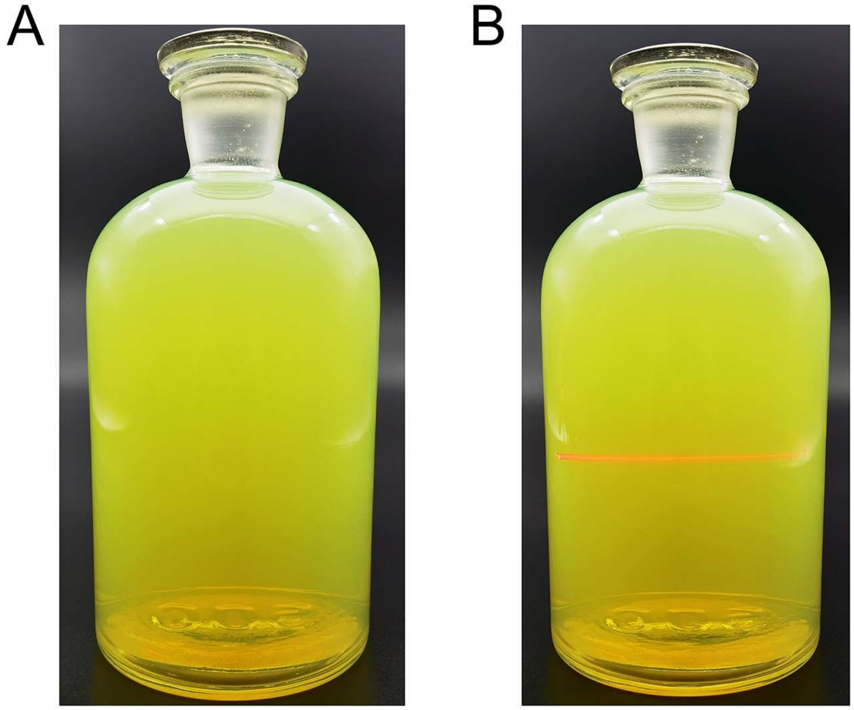

Figure S3. (A) Digital photograph and (B) Tyndall effect of the N-$ZnIn_2S_4$ colloidal solution (2 mg mL^{-1}, 500 mL).

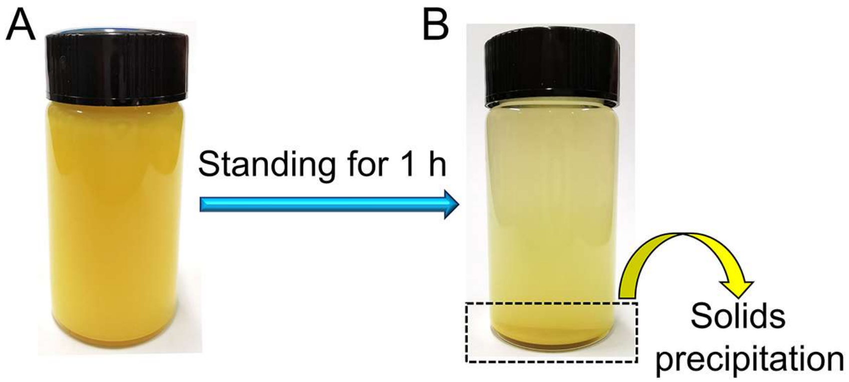

Figure S4. Digital photograph of (A) the F-$ZnIn_2S_4$ suspension after ultrasonic treatment for 3 h, and (B) F-$ZnIn_2S_4$ sediment after standing for 1 h.

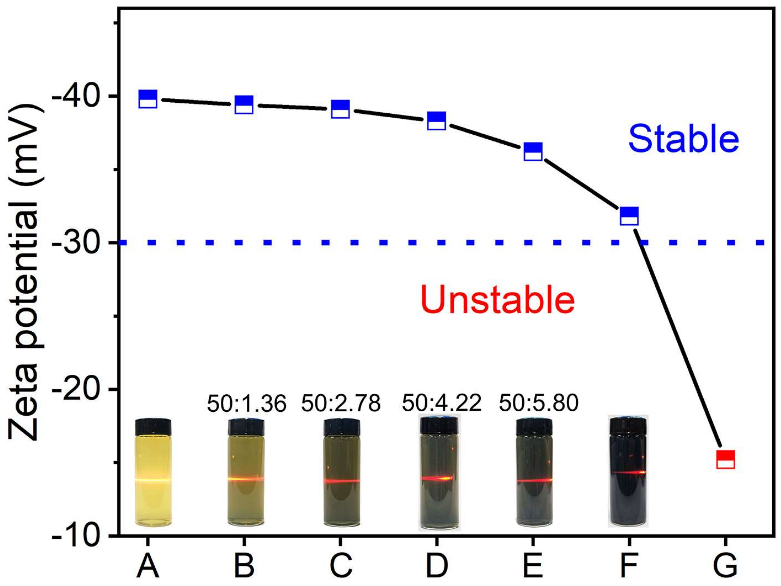

Figure S5. Zeta potential of (A) N-$ZnIn_2S_4$ colloidal solution, (B, C, D, E) mixed colloidal solution with different volume ratio (mL: mL) of N-$ZnIn_2S_4$/$Ti_3C_2T_x$, (F) $Ti_3C_2T_x$ colloidal solution, and (G) 4-TC/N-ZIS suspension solution. The insets showed the Tyndall effect of the colloidal solution.

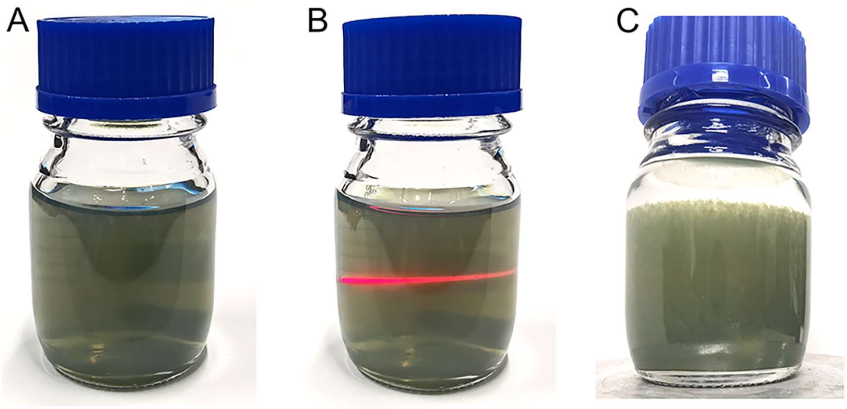

Figure S6. (A) Digital photograph and the (B) Tyndall effect of the mixed $Ti_3C_2T_x$/N-$ZnIn_2S_4$ colloid solution of with 4 wt.% $Ti_3C_2T_x$ relative to N-$ZnIn_2S_4$, (C) suspension solution of the $Ti_3C_2T_x$/N-$ZnIn_2S_4$ composites formed by adding the NH_4HCO_3.

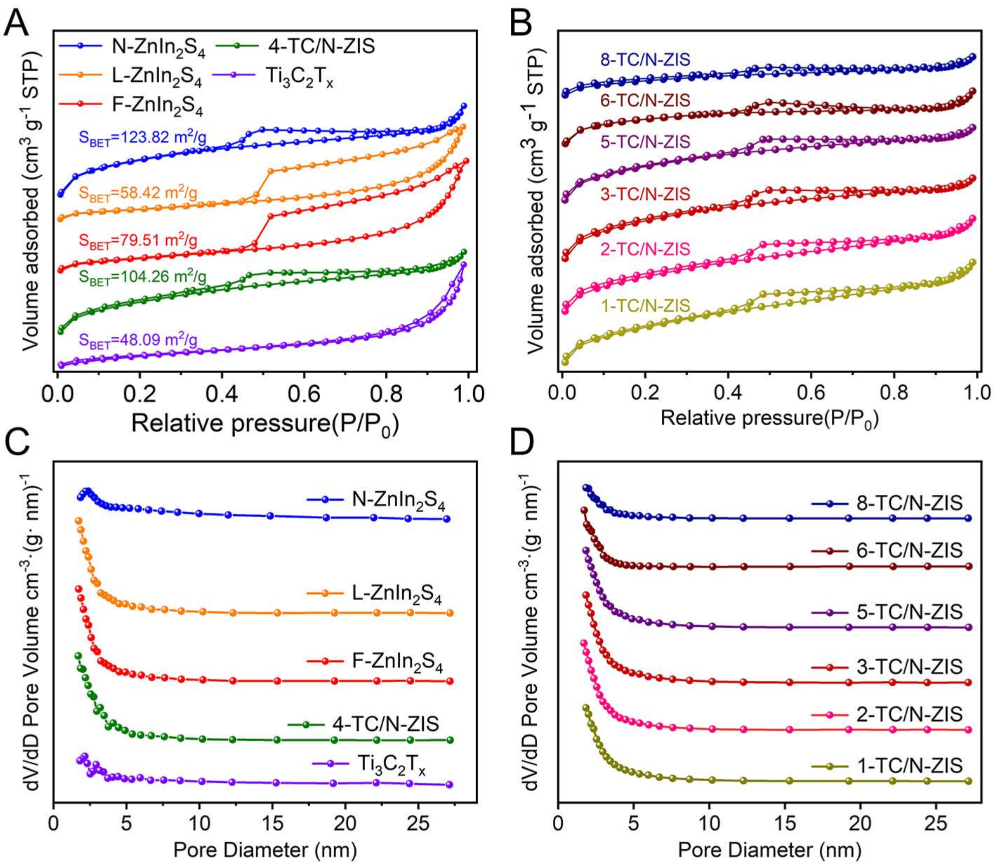

Figure S7. N_2 adsorption-desorption isotherms of (A) F-$ZnIn_2S_4$, L-$ZnIn_2S_4$, N-$ZnIn_2S_4$, $Ti_3C_2T_x$, and 4-TC/N-ZIS and (B) 1-TC/N-ZIS, 2-TC/N-ZIS, 3-TC/N-ZIS, 5-TC/N-ZIS, 6-TC/N-ZIS, and 8-TC/N-ZIS. (C) Pore size distribution of F-$ZnIn_2S_4$, L-$ZnIn_2S_4$, N-$ZnIn_2S_4$, $Ti_3C_2T_x$, and 4-TC/N-ZIS. (D) Pore size distribution of 1-TC/N-ZIS, 2-TC/N-ZIS, 3-TC/N-ZIS, 5-TC/N-ZIS, 6-TC/N-ZIS, and 8-TC/N-ZIS.

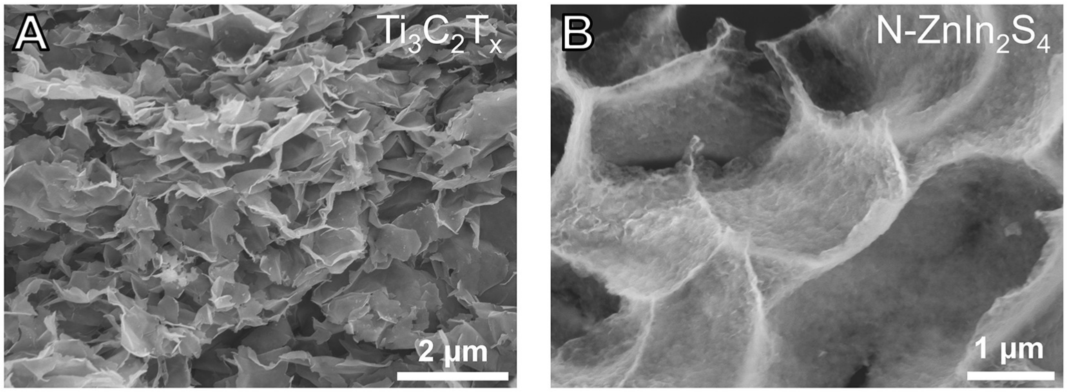

Figure S8. SEM images of (A) $Ti_3C_2T_x$ and (B) N-$ZnIn_2S_4$.

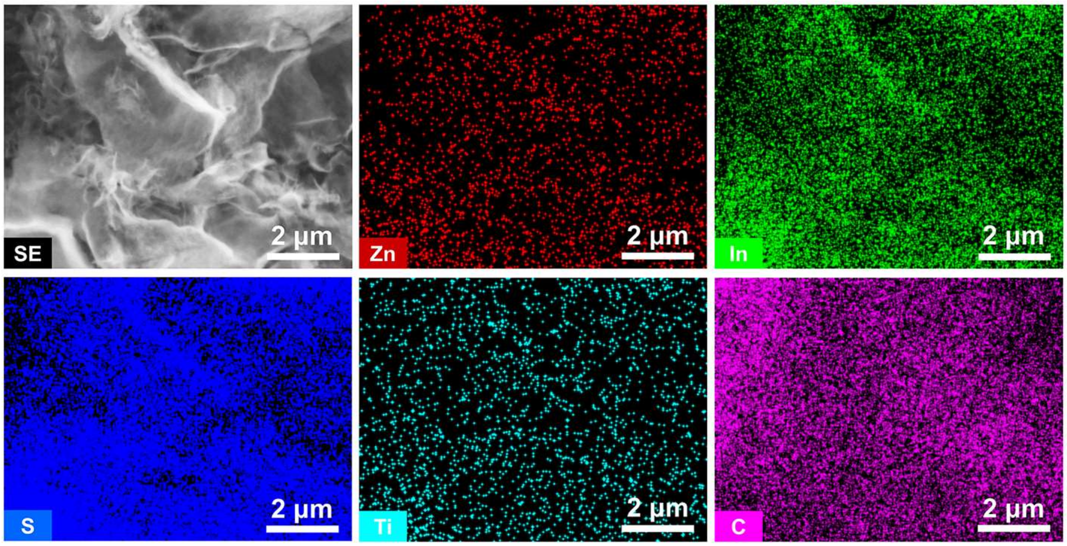

Figure S9. SEM image and the corresponding EDS element mapping of 4-TC/N-ZIS.

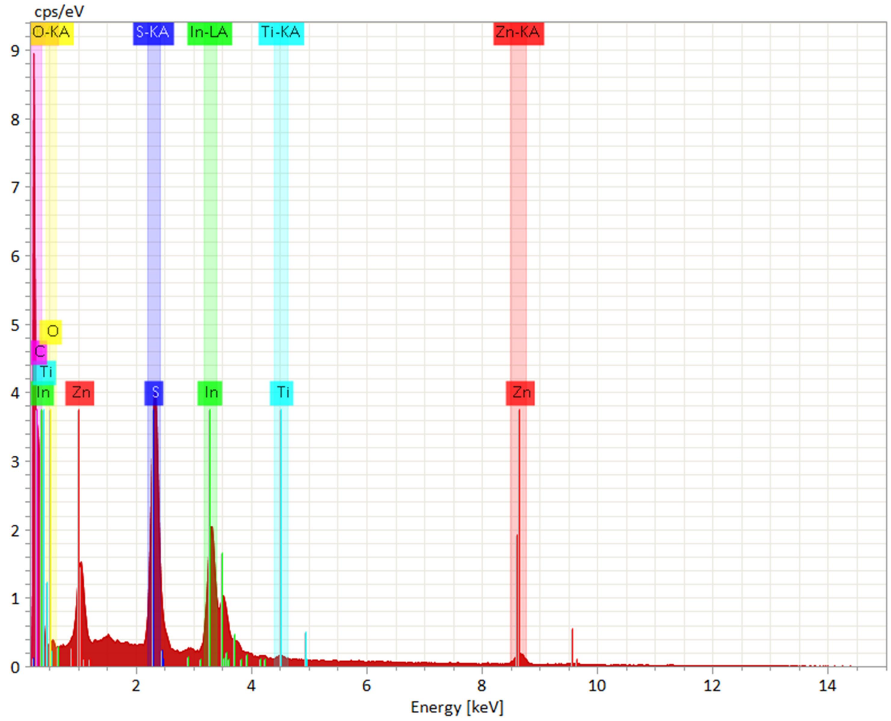

Figure S10. EDS spectrum of 4-TC/N-ZIS.

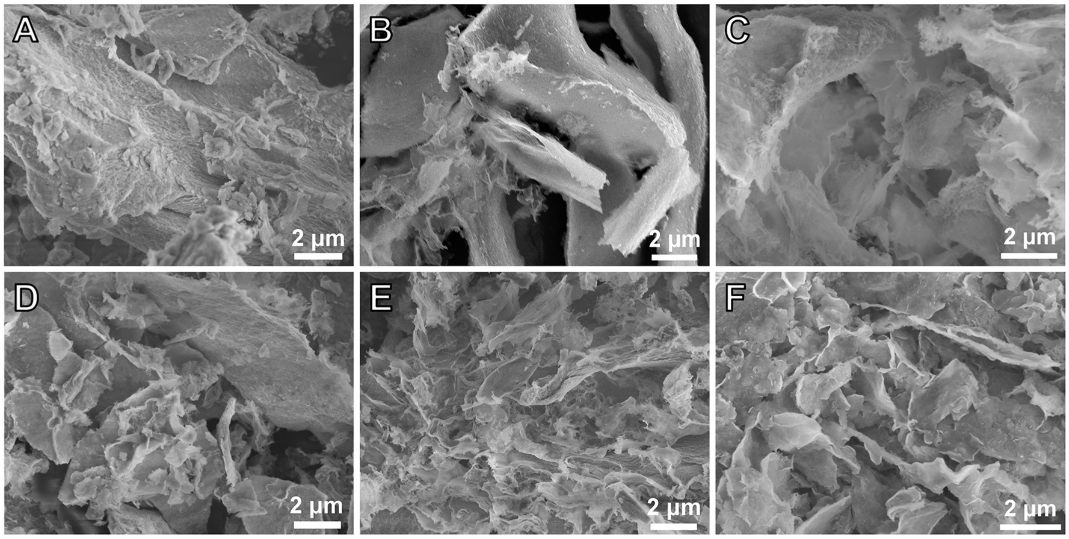

Figure S11. SEM images of (A) 1-TC/N-ZIS, (B) 2-TC/N-ZIS, (C) 3-TC/N-ZIS, (D) 5-TC/N-ZIS, (E) 6-TC/N-ZIS, and (F) 8-TC/N-ZIS.

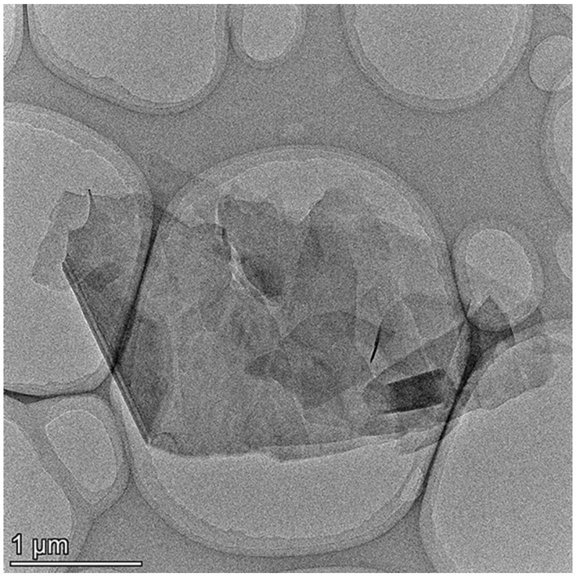

Figure S12. TEM image of $Ti_3C_2T_X$ on the copper grid.

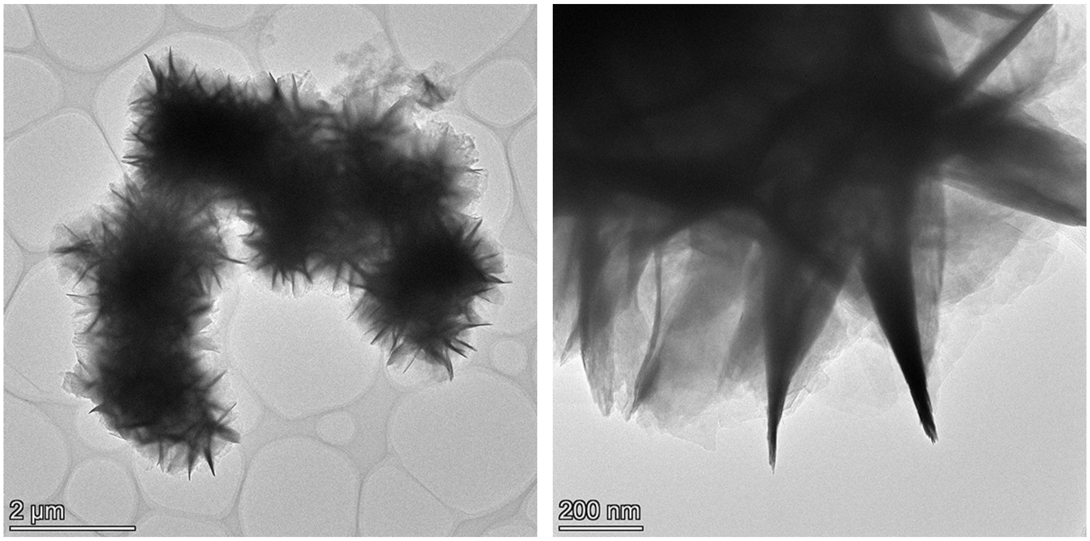

Figure S13. TEM images of F-$ZnIn_2S_4$.

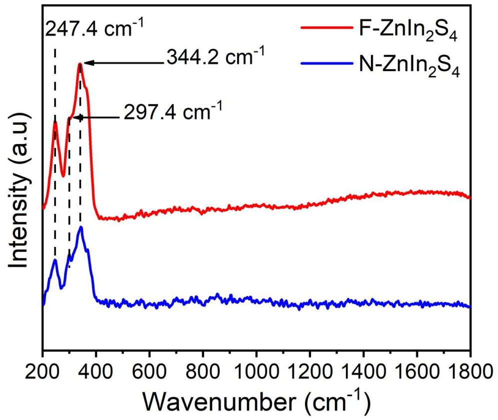

Figure S14. Raman spectra of F-$ZnIn_2S_4$ and N-$ZnIn_2S_4$.

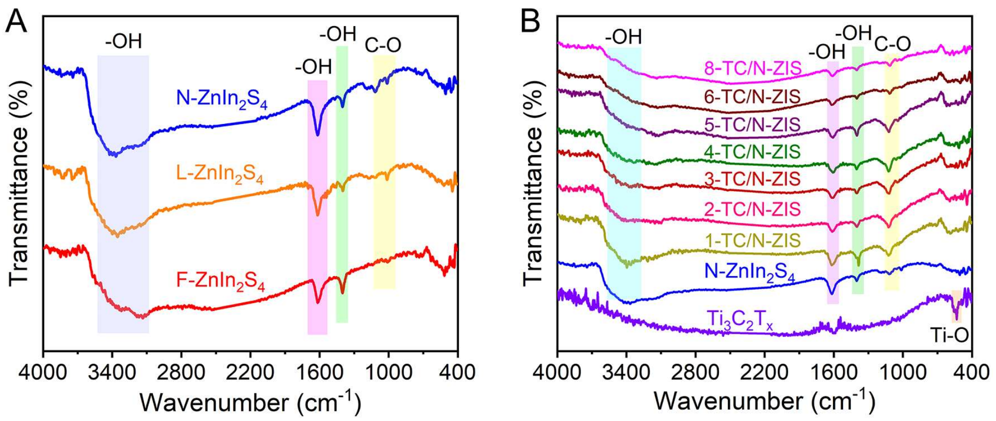

Figure S15. FT-IR spectra of (A) F-$ZnIn_2S_4$, L-$ZnIn_2S_4$, N-$ZnIn_2S_4$, (B) $Ti_3C_2T_x$, and x-TC/N-ZIS (x = 1, 2, 3, 4, 5, 6 and 8).

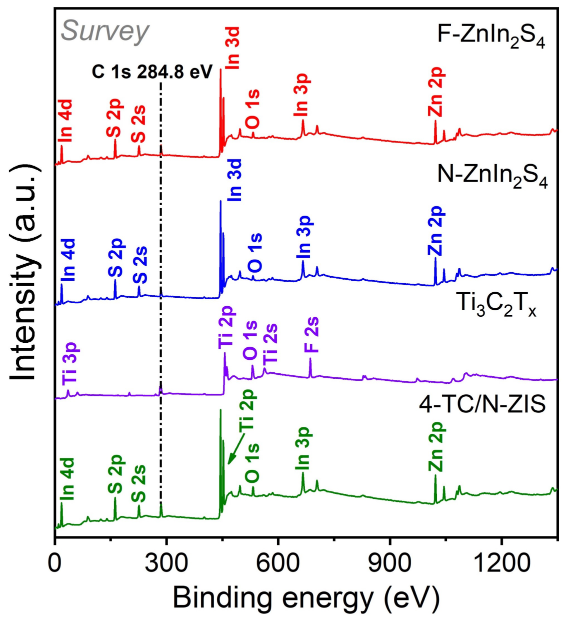

Figure S16. (A) XPS survey spectra of F-$ZnIn_2S_4$, N-$ZnIn_2S_4$, $Ti_3C_2T_x$ and 4-TC/ZIS.

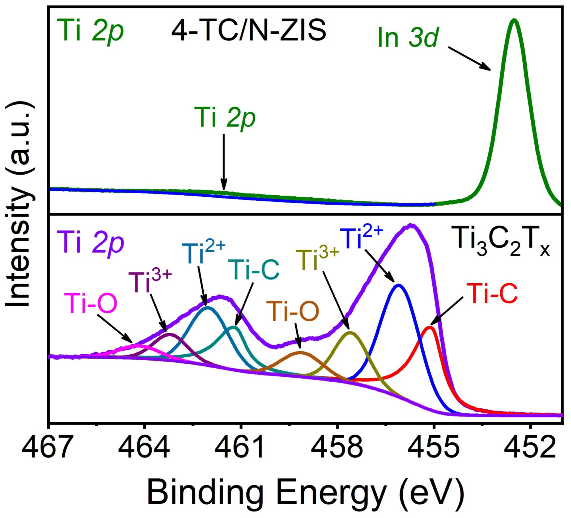

Figure S17. High-resolution XPS peak deconvolution of Ti 2p in $Ti_3C_2T_x$ and 4-TC/N-ZIS.

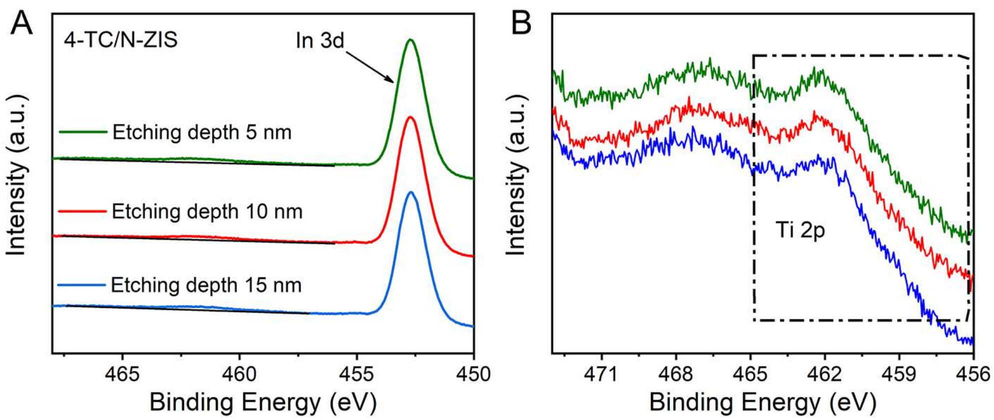

Figure S18. High-resolution XPS peak deconvolution of Ti 2p in 4-TC/N-ZIS after Ar ion etching at different depths of 5 nm, 10 nm , and 15 nm.

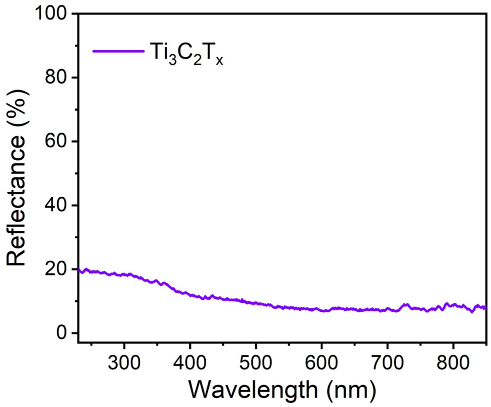

Figure S19. Ultraviolet-visible diffuse reflectance spectra of $Ti_3C_2T_x$.

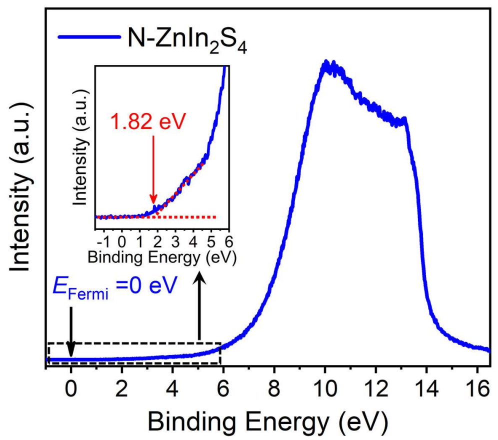

Figure S20. UPS valence band spectrum of N-ZIS after Fermi level correction.

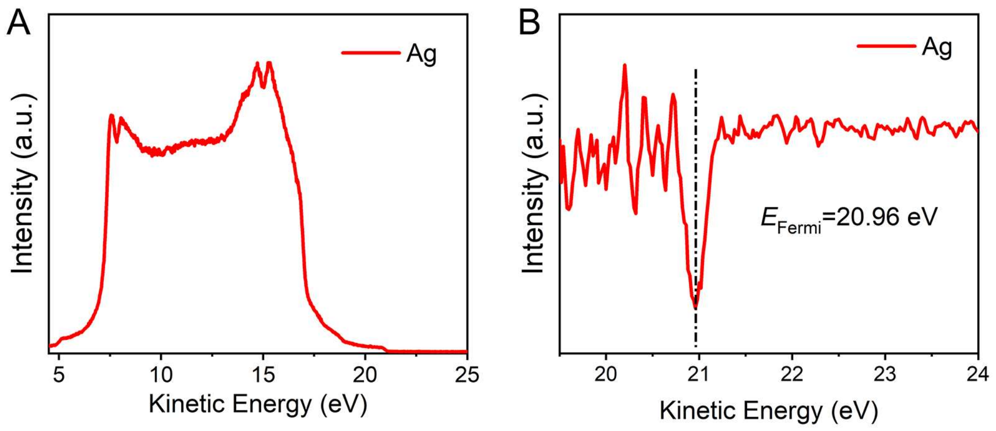

Figure S21. The UPS spectrum and Fermi energy level of the Ag standard sample (without adding bias voltage).

The energy spectrum was obtained by using 21.22 eV excitation source without adding bias voltage. The Fermi edge kinetic energy of Ag standard sample was 20.96 eV, thus, the correction value was 0.26 eV for UPS spectrum of valence band.

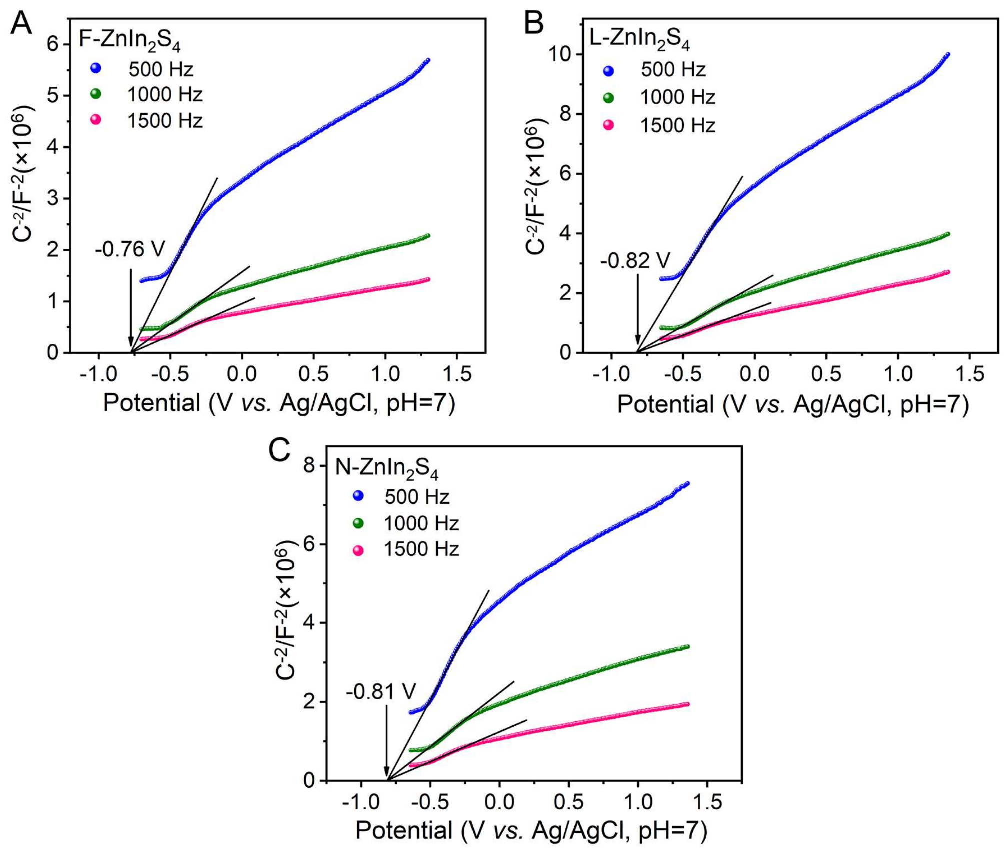

Figure S22. Mott-Schottky plots at different frequencies (500 Hz, 1000 Hz, 1500 Hz) for (A) F-$ZnIn_2S_4$, (B) L-$ZnIn_2S_4$, and (C) N-$ZnIn_2S_4$.

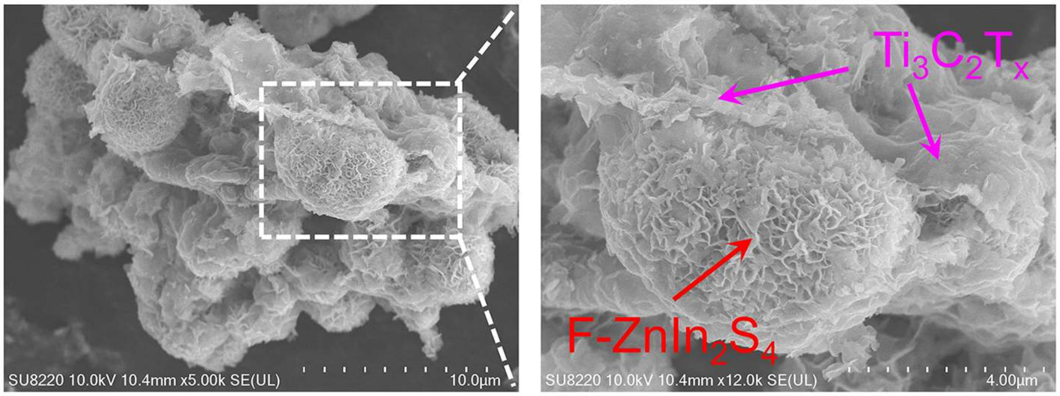

Figure S23. SEM images of 4-TC/F-ZIS.

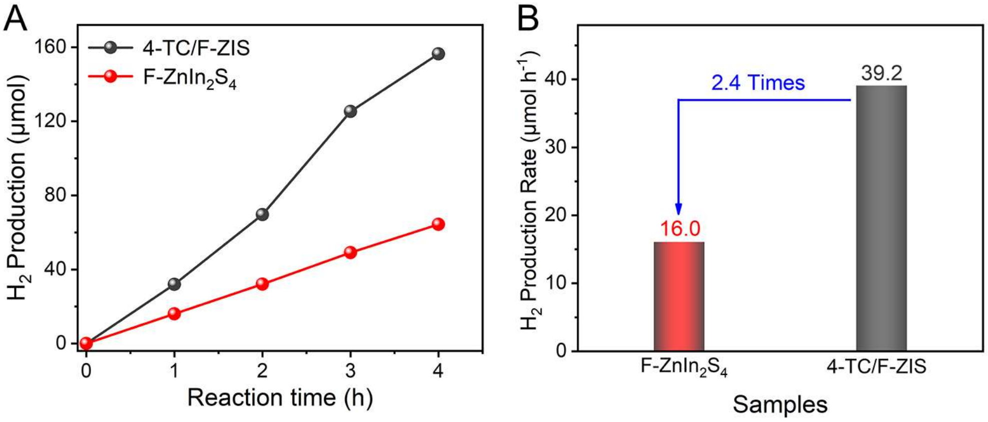

Figure S24. (A) Time course of photocatalytic H_2 production performance and (B) the photocatalytic H_2 production rate of F-$ZnIn_2S_4$ and 4-TC/F-ZIS.

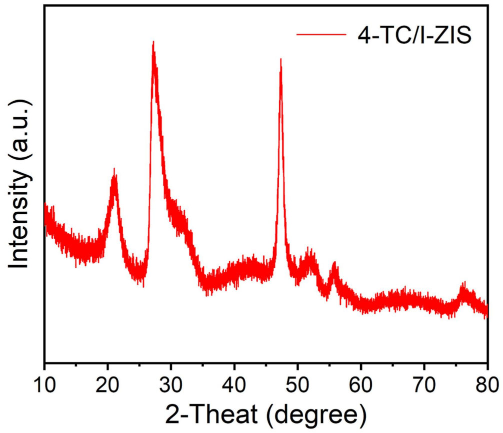

Figure S25. XRD pattern of 4-TC/I-ZIS.

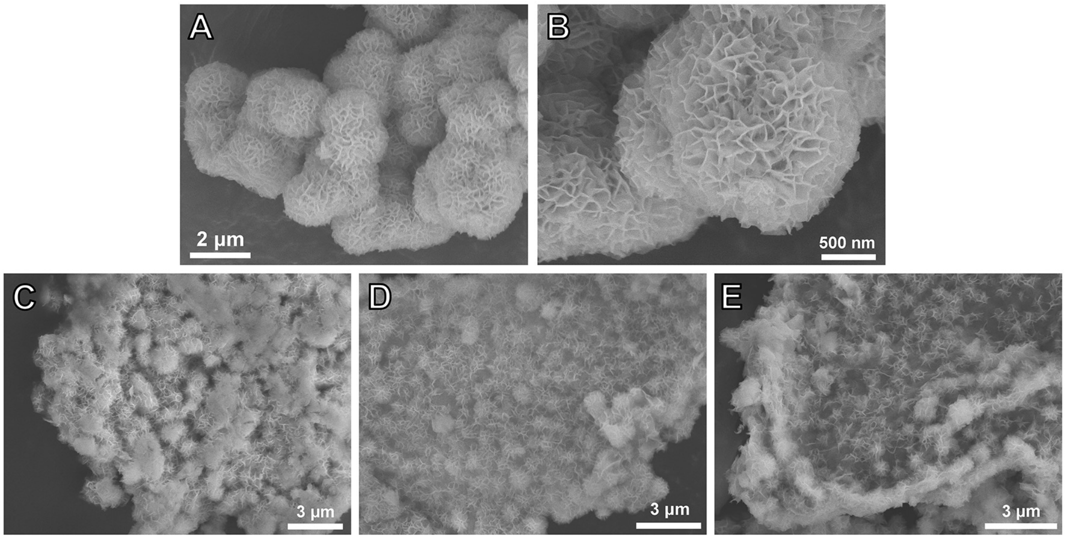

Figure S26. SEM images of (A-B) I-$ZnIn_2S_4$, (C) 2-TC/I-ZIS, (D) 4-TC/I-ZIS, and (E) 6-TC/I-ZIS.

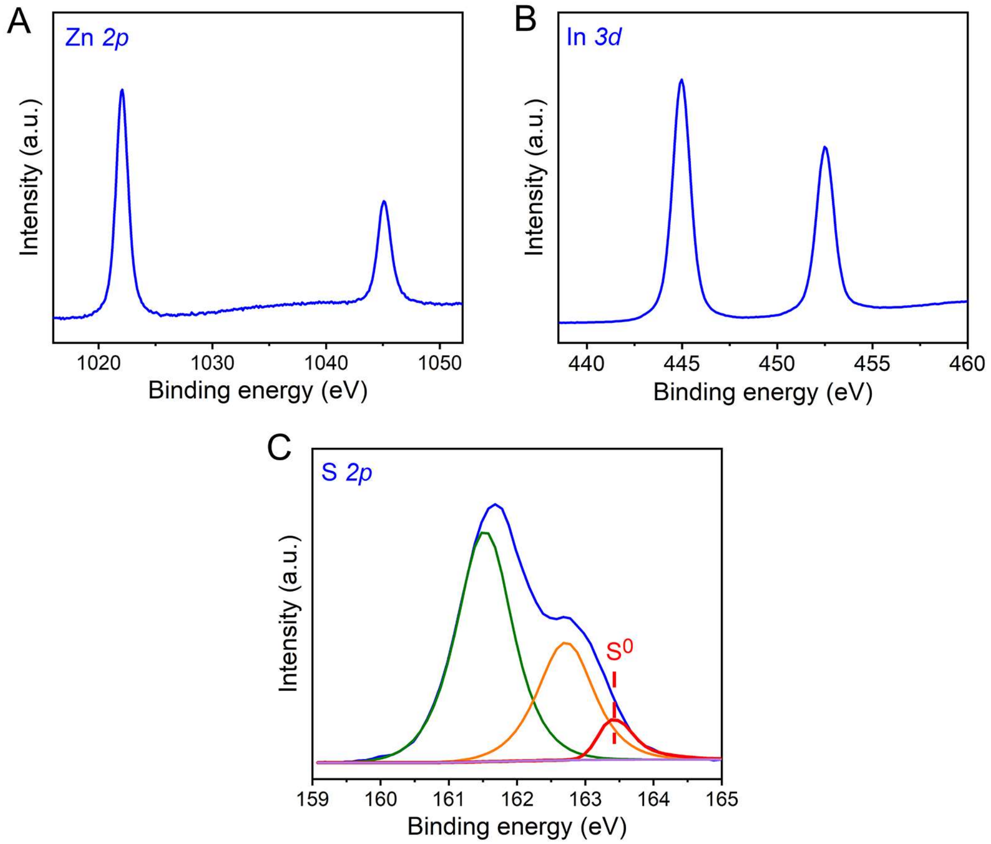

Figure S27. High-resolution XPS spectra of (A) Zn 2p, (B) In 3d, and (C) S 2p in N-$ZnIn_2S_4$ after reaction.

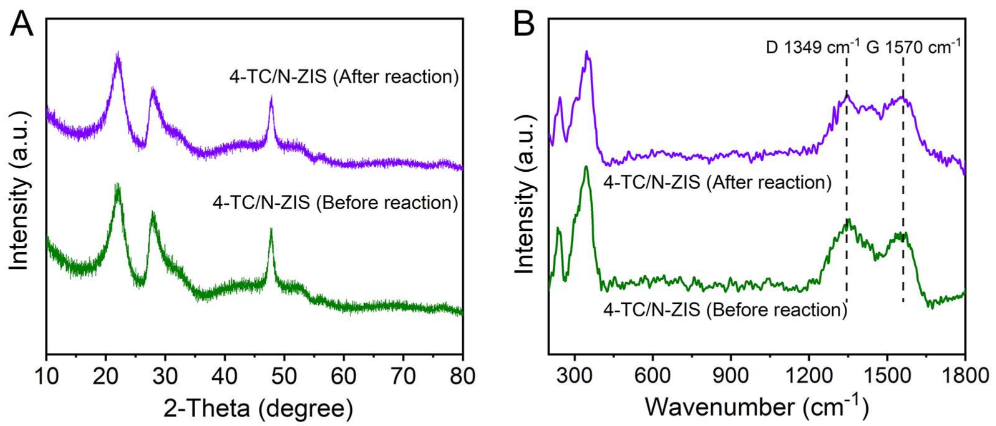

Figure S28. (A) XRD patterns and (B) Raman spectra of 4-TC/N-ZIS before and after reaction.

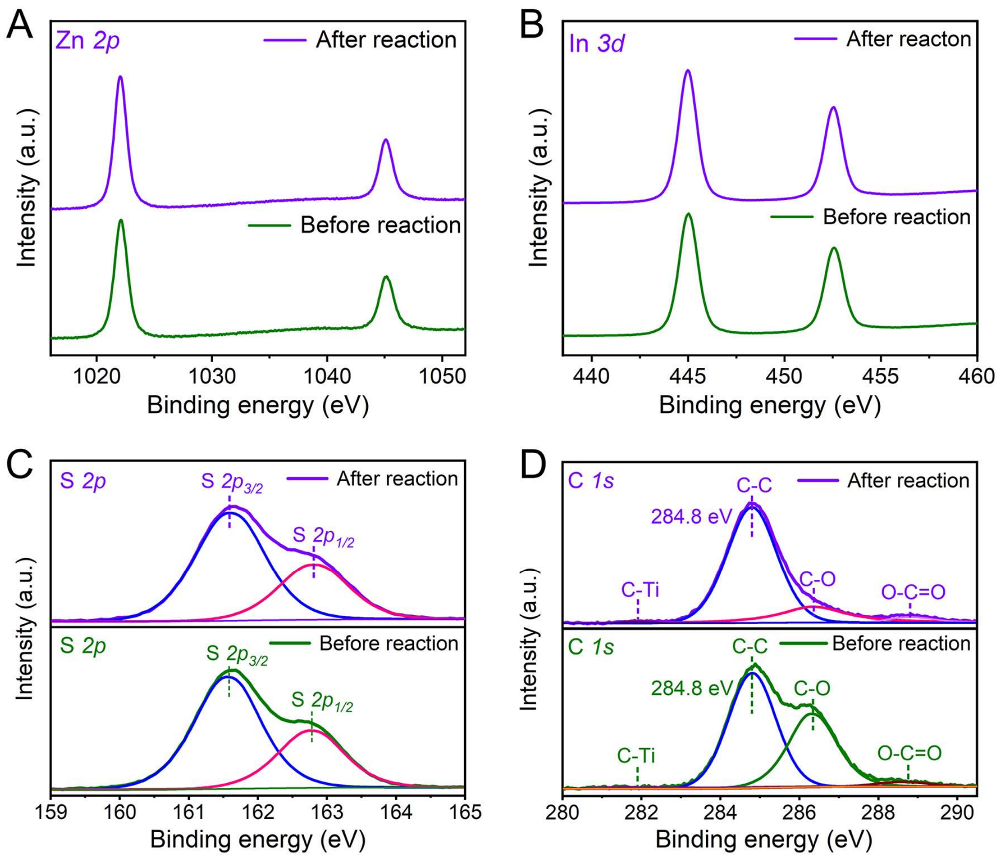

Figure S29. High-resolution XPS spectrum of (A) Zn 2p, (B) In 3d , (C) S 2p, and (D) C 1s in 4-TC/N-ZIS before and after reaction.

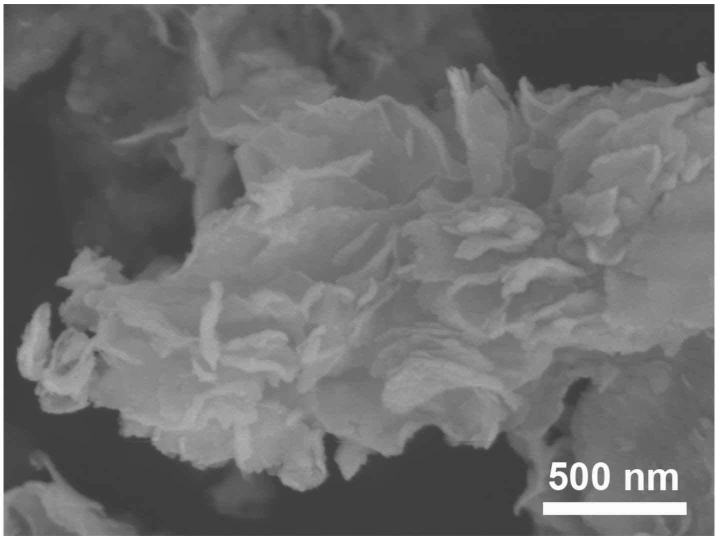

Figure S30. SEM image of 4-TC/N-ZIS after reaction.

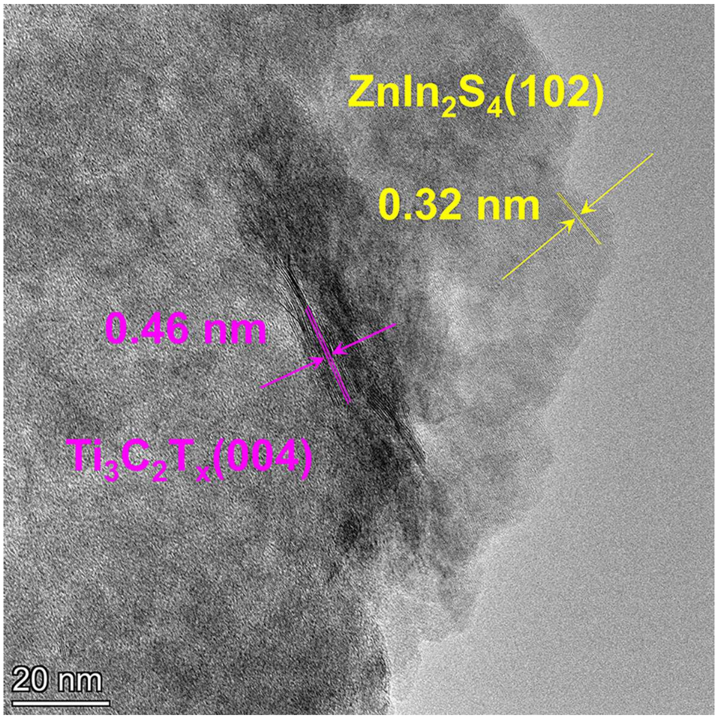

Figure S31. HRTEM image of 4-TC/N-ZIS after reaction.

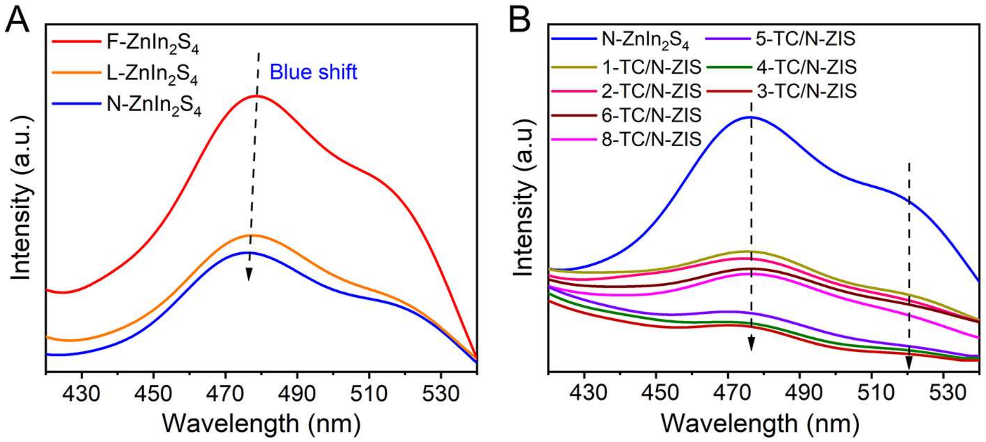

Figure S32. Steady-state PL spectra of (A) F-$ZnIn_2S_4$, L-$ZnIn_2S_4$, N-$ZnIn_2S_4$, and (B) x-TC/N-ZIS (x = 1, 2, 3, 4, 5, 6, 8),

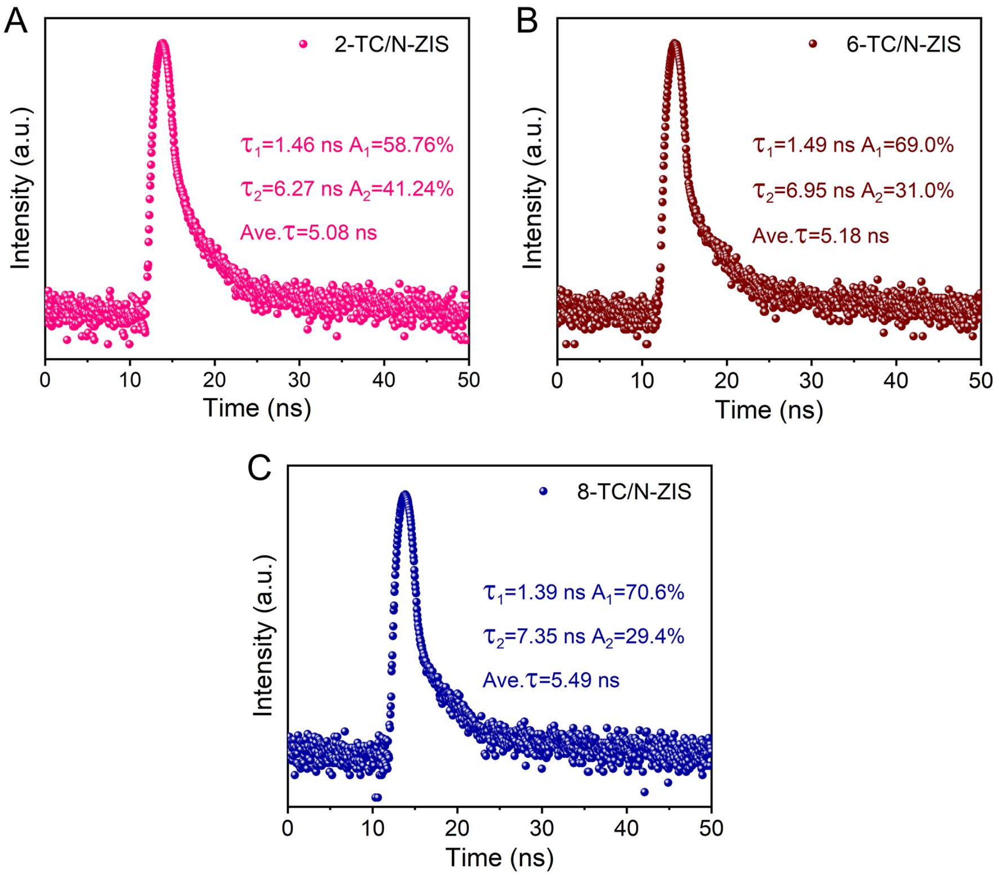

Figure S33. Time-resolved PL spectra of (A) 2-TC/N-ZIS, (B) 6-TC/N-ZIS, and (C) 8-TC/N-ZIS.

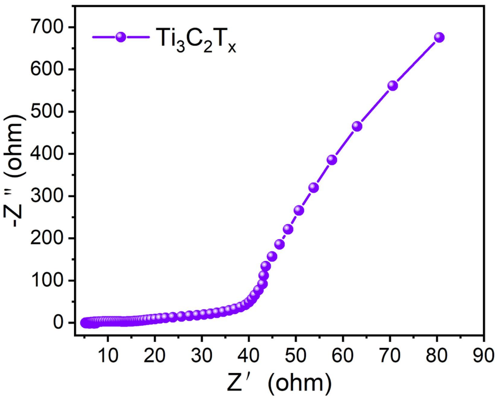

Figure S34. EIS Nyquist plots of $Ti_3C_2T_x$.

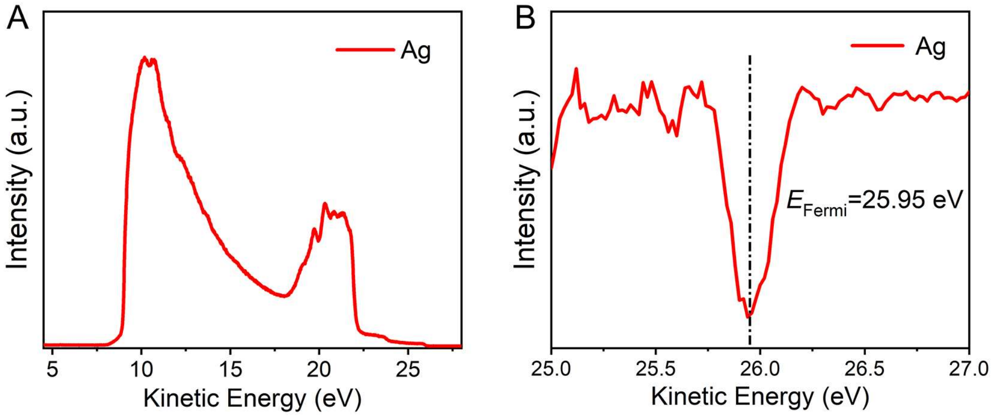

Figure S35. The UPS spectrum and Fermi energy level of the Ag standard sample (with adding -5 eV bias voltage).

The spectra spectrum was obtained by using 21.22 eV excitation source with a bias voltage of -5 eV. The Fermi edge kinetic energy of Ag standard sample was 25.95 eV, thus, the actual bias voltage was -4.73 eV.

Table S1. Surface area and pore diameter of F-$ZnIn_2S_4$, L-$ZnIn_2S_4$, N-$ZnIn_2S_4$, $Ti_3C_2T_x$ and x-TC/N-ZIS.

Samples	BET surface area ($m^2 \cdot g^{-1}$)	Pore diameter (nm)
F-$ZnIn_2S_4$	79.51	6.18
L-$ZnIn_2S_4$	58.42	10.43
N-$ZnIn_2S_4$	123.82	4.30
$Ti_3C_2T_x$	48.09	11.54
1-TC/N-ZIS	80.01	3.86
2-TC/N-ZIS	83.40	3.37
3-TC/N-ZIS	90.58	3.64
4-TC/N-ZIS	104.26	3.65
5-TC/N-ZIS	81.02	3.70
6-TC/N-ZIS	60.53	4.01
8-TC/N-ZIS	55.73	4.04

Table S2. Elemental analysis of the 4-TC/N-ZIS photocatalyst by EDS.

Element	Atomic (%)
Zn	4.21
In	7.45
S	13.43
Ti	0.31
C	66.29
O	8.31
$ZnIn_{1.77}S_{3.19}$	

Table S3. The I_D/I_G value of $Ti_3C_2T_x$ and x-TC/N-ZIS (x = 1, 2, 3, 4, 5, 6, 8) in Raman spectra.

Samples	I_D/I_G
$Ti_3C_2T_x$	0.69
1-TC/N-ZIS	0.72
2-TC/N-ZIS	1.31
3-TC/N-ZIS	1.20
4-TC/N-ZIS	1.28
5-TC/N-ZIS	1.53
6-TC/N-ZIS	1.41
8-TC/N-ZIS	1.48

Table S4. The calculated hydrogen production rate of F-$ZnIn_2S_4$, L-$ZnIn_2S_4$, N-$ZnIn_2S_4$, $Ti_3C_2T_x$, and x-TC/N-ZIS divided by specific surface area.

Samples	BET surface area (m^2 g^{-1})	H_2 Production Rate (mmol g^{-1} h^{-1})	H_2 Production Rate (μmol h^{-1} m^{-2})
F-$ZnIn_2S_4$	79.51	0.80	10.06
L-$ZnIn_2S_4$	58.42	1.12	19.17
N-$ZnIn_2S_4$	123.82	2.04	16.48
$Ti_3C_2T_x$	48.09	0	0
1-TC/N-ZIS	80.01	3.45	43.12
2-TC/N-ZIS	83.40	4.37	52.40
3-TC/N-ZIS	90.58	5.85	64.58
4-TC/N-ZIS	104.26	7.42	71.17
5-TC/N-ZIS	81.02	5.36	66.16
6-TC/N-ZIS	60.53	3.34	55.18
8-TC/N-ZIS	55.73	3.12	55.98

Table S5. Comparison the photocatalytic hydrogen evolution performance of $ZnIn_2S_4$-based photocatalyst in recent reports.

Photocatalyst	Light source	Sacrificial agents/cocatalyst	Yield of H_2 ($mmol \cdot g^{-1} \cdot h^{-1}$)	AQE（%）	Ref.
Co_9S_8@$ZnIn_2S_4$	300 W Xe lamp, λ≥400 nm	TEOA	6.250	None	[2]
$Ti_3C_2T_x/ZnIn_2S_4$	300 W Xe lamp, λ≥400 nm	TEOA/3%Pt	6.482	20.41 (λ=400 nm)	[1a]
$Ni_{1-x}Co_xSe_2C$ $/ZnIn_2S_4$	300 W Xe lamp, λ≥420 nm	TEOA	5.099	2.32 (λ=420 nm)	[1b]
$CdS/ZnIn_2S_4$	300 W Xe lamp, λ: 320 - 780 nm	Na_2S/Na_2SO_3	3.072	15.9 (λ=420 nm)	[3]
$ZnIn_2S_4/g\text{-}C_3N_4$	300 W Xe lamp, λ≥400 nm	TEOA/1%Pt	4.854	None	[4]
NH_2-MIL-125(Ti) @$ZnIn_2S_4$/CdS	300 W Xe lamp, λ≥400 nm	lactic acid	2.367	None	[5]
$Ag_{0.6}Au_{0.4}/ZnIn_2S_4$	300 W Xe lamp	Na_2S/Na_2SO_3	5.401	8.6	[6]
N–$La_2Ti_2O_7$ $/ZnIn_2S_4$	300 W Xe lamp	lactic acid	4.46	20.79 (λ=435 nm)	[7]
Ti_3C_2@TiO_2 $/ZnIn_2S_4$	300 W Xe lamp	Na_2S/Na_2SO_3	1.186	None	[8]
UiO-66/$ZnIn_2S_4$	300 W Xe lamp, λ≥400 nm	TEOA	3.062	19.39 (λ=400 nm)	[9]

Photocatalyst	Light source	Sacrificial agents/cocatalyst	Yield of H_2 ($mmol\cdot g^{-1}\cdot h^{-1}$)	AQE（%）	Ref.
$WO_{3-x}/ZnIn_2S_4$	300 W Xe lamp, λ≥400 nm	TEOA	5.769	26.28 (λ=400 nm)	[10]
$ZnIn_2S_4@Co_3S_4$	300 W Xe lamp, λ≥350 nm	TEOA	4.261	None	[11]
$ZnIn_2S_4/BiVO_4$	300 W Xe lamp, λ≥400 nm	TEOA/3%Pt	5.944	8.15 (λ=405 nm)	[12]
$CoFe_2O_4/ZnIn_2S_4$	300 W Xe lamp, λ≥400 nm	TEOA/3%Pt	0.8	5.0 (λ=420 nm)	[13]
$ZnIn_2S_4/MoSe_2$	300 W Xe lamp, λ≥400 nm	lactic acid	6.454	None	[14]
$ReS_2/ZnIn_2S_4$	300 W Xe lamp, λ≥420 nm	TEOA	1.859	None	[15]
$ZnIn_2S_4$ /S,N-codoped carbon	300 W Xe lamp	TEOA	2.937	19.47 (λ=435 nm)	[16]
MoS_2QDs@Vs-M-$ZnIn_2S_4$	300 W Xe lamp, λ:320-780 nm	lactic acid	6.884	63.87 (λ=420 nm)	[17]
Ti_3C_2/S-vacancy $ZnIn_2S_4$	300 W Xe lamp, λ≥400 nm	TEOA	7.42	12.84 (λ=400 nm)	This work
Ti_3C_2/S-vacancy $ZnIn_2S_4$	300 W Xe lamp, λ≥400 nm	TEOA/3%Pt	19.23	28.61 (λ=400 nm)	This work

References

[1] a) G. Zuo, Y. Wang, W. L. Teo, A. Xie, Y. Guo, Y. Dai, W. Zhou, D. Jana, Q. Xian, W. Dong, Y. Zhao, *Angewandte Chemie International Edition* **2020**, *59* (28), 11287; b) Y. Chao, P. Zhou, J. Lai, W. Zhang, H. Yang, S. Lu, H. Chen, K. Yin, M. Li, L. Tao, C. Shang, M. Tong, S. Guo, *Advanced Functional Materials* **2021**, *31* (24), 2100923.

[2] S. Wang, B. Y. Guan, X. Wang, X. W. D. Lou, *Journal of the American Chemical Society* **2018**, *140* (45), 15145.

[3] Y. Zhu, J. Chen, L. Shao, X. Xia, Y. Liu, L. Wang, *Applied Catalysis B: Environmental* **2020**, *268*, 118744.

[4] Z. Gao, K. Chen, L. Wang, B. Bai, H. Liu, Q. Wang, *Applied Catalysis B: Environmental* **2020**, *268*, 118462.

[5] S. Zhang, M. Du, Z. Xing, Z. Li, K. Pan, W. Zhou, *Applied Catalysis B: Environmental* **2020**, *262*, 118202.

[6] H. An, M. Li, R. Liu, Z. Gao, Z. Yin, *Chemical Engineering Journal* **2020**, *382*, 122953.

[7] M. Xia, X. Yan, H. Li, N. Wells, G. Yang, *Nano Energy* **2020**, *78*, 105401.

[8] K. Huang, C. Li, X. Meng, *Journal of Colloid and Interface Science* **2020**, *580*, 669.

[9] X. Peng, L. Ye, Y. Ding, L. Yi, C. Zhang, Z. Wen, *Applied Catalysis B: Environmental* **2020**, *260*, 118152.

[10] D. Luo, L. Peng, Y. Wang, X. Lu, C. Yang, X. Xu, Y. Huang, Y. Ni, *Journal of Materials Chemistry A* **2021**, *9* (2), 908.

[11] G. Wang, W. Chen, Y. Zhang, Q. Xu, Y. Li, M. L. Foo, L. Tang, *RSC Advances* **2021**, *11* (16), 9296.

[12] J. Hu, C. Chen, Y. Zheng, G. Zhang, C. Guo, C. M. Li, *Small* **2020**, *16* (37), 2002988.

[13] C. Li, H. Che, P. Huo, Y. Yan, C. Liu, H. Dong, *Journal of Colloid and Interface Science* **2021**, *581*, 764.

[14] M.-Q. Yang, Y.-J. Xu, W. Lu, K. Zeng, H. Zhu, Q.-H. Xu, G. W. Ho, *Nature Communications* **2017**, *8* (1), 14224.

[15] X. Xiong, A. Yan, X. Zhang, F. Huang, Z. Li, Z. Zhang, H. Weng, *Journal of Alloys and Compounds* **2021**, *873*, 159850.

[16] X. Fu, C. Huang, J. Wen, Y. Du, X. Zheng, *International Journal of Hydrogen Energy* **2021**, *46* (34), 17697.

[17] S. Zhang, X. Liu, C. Liu, S. Luo, L. Wang, T. Cai, Y. Zeng, J. Yuan, W. Dong, Y. Pei, Y. Liu, *ACS Nano* **2018**, *12* (1), 751.
